# Evolutionary compromises in fungal fitness: hydrophobins can hinder the adverse dispersal of conidiospores and challenge their survival

**DOI:** 10.1038/s41396-020-0709-0

**Published:** 2020-07-06

**Authors:** Feng Cai, Renwei Gao, Zheng Zhao, Mingyue Ding, Siqi Jiang, Civan Yagtu, Hong Zhu, Jian Zhang, Thomas Ebner, Michael Mayrhofer-Reinhartshuber, Philipp Kainz, Komal Chenthamara, Günseli Bayram Akcapinar, Qirong Shen, Irina S. Druzhinina

**Affiliations:** 1 The Key Laboratory of Plant Immunity, Jiangsu Provincial Key Lab of Solid Organic Waste Utilization, Nanjing Agricultural University, 210095 Nanjing, China; 2 Fungal Genomics Laboratory (FungiG), Nanjing Agricultural University, 210095 Nanjing, China; 3 Institute of Chemical, Environmental and Bioscience Engineering (ICEBE), TU Wien, A1060 Vienna, Austria; 4 KML Vision GmbH, A8020 Graz, Austria; 5 Department of Medical Biotechnology, Institute of Health Sciences, Acibadem Mehmet Ali Aydinlar University, Istanbul, Turkey

**Keywords:** Fungal ecology, Microbial ecology, Molecular evolution

## Abstract

Fungal evolutionary biology is impeded by the scarcity of fossils, irregular life cycles, immortality, and frequent asexual reproduction. Simple and diminutive bodies of fungi develop inside a substrate and have exceptional metabolic and ecological plasticity, which hinders species delimitation. However, the unique fungal traits can shed light on evolutionary forces that shape the environmental adaptations of these taxa. Higher filamentous fungi that disperse through aerial spores produce amphiphilic and highly surface-active proteins called hydrophobins (HFBs), which coat spores and mediate environmental interactions. We exploited a library of HFB-deficient mutants for two cryptic species of mycoparasitic and saprotrophic fungi from the genus *Trichoderma* (Hypocreales) and estimated fungal development, reproductive potential, and stress resistance. HFB4 and HFB10 were found to be relevant for *Trichoderma* fitness because they could impact the spore-mediated dispersal processes and control other fitness traits. An analysis in silico revealed purifying selection for all cases except for HFB4 from *T. harzianum*, which evolved under strong positive selection pressure. Interestingly, the deletion of the *hfb4* gene in *T. harzianum* considerably increased its fitness-related traits. Conversely, the deletion of *hfb4* in *T. guizhouense* led to the characteristic phenotypes associated with relatively low fitness. The net contribution of the *hfb4* gene to fitness was found to result from evolutionary tradeoffs between individual traits. Our analysis of HFB-dependent fitness traits has provided an evolutionary snapshot of the selective pressures and speciation process in closely related fungal species.

## Introduction

Ubiquitously spread on land and ocean, fungi form one of the most diverse eukaryotic kingdoms with millions of species [1, 2]. However, the peculiarities of their biology such as their pleomorphic life cycles with frequent prevalence of asexual reproduction, prolonged stages of dormancy, and superior metabolic plasticity impede studies on fungal evolution and ecology, leaving this group largely unexplored [3].

Fungal systematics is challenged by complexes of cryptic species [4], which are morphologically identical taxa that can only be distinguished based on genetic data [5]. Recent genomic studies point to considerable genetic distances between such species [6, 7], but the evolutionary forces driving fungal speciation remain poorly understood [4, 5]. Consequently, fungi are rarely investigated for ecological genetics—i.e., for genes that are relevant to their fitness [8].

In asexually reproducing fungi with a haploid life cycle, fitness can be directly assigned to a haplotype [8, 9], but there is no consensus on how to measure the fitness of these organisms [9]. Currently, only two aspects of fungal life cycle are widely accepted as fitness metrics for filamentous fungi: mycelial growth rate and the amount of produced spores [8]. However, growth and sporulation do not guarantee reproductive success. Mature spores need to be discharged in the optimal conditions for efficient dispersal through air and water or by animals [10, 11]. When spores are carried over a long distance, survival is compromised by exposure to diverse abiotic stressors, such as drought, extreme temperatures, and ultraviolet (UV) radiation [12, 13]. Thus, fitness can be estimated using not only developmental parameters but also spore properties that influence dispersal efficiency in different media and resistance to stressors.

Most filamentous fungi disperse through airborne spores that can be transferred by wind over a long distance [11, 14]. For this reason, fungal spores usually remain dry and hydrophobic [15]. The hydrophobicity of spores is thought to be provided by hydrophobins (HFBs), which are small secreted cysteine-rich proteins (usually <20 kDa) that are characterized by a conserved pattern of eight cysteines (Cys) [16]. HFBs are only known from higher filamentous fungi (Dikarya) and reported to be some of the most surface-active proteins in nature [15, 17]. Previous work indicates that HFBs are secreted in a soluble form and spontaneously localize and self-organize at hydrophilic-hydrophobic interfaces, where they assemble into insoluble, amphipathic elastic layers [17, 18]. These layers cover fungal bodies and spores in water-repelling coats [17, 19] and influence spore dispersal [20], stress resistance, development, and biotic interactions [21, 22].

In pathogenic fungi such as *Aspergillus fumigatus* (Eurotiales) [15], Ma*gnaporthe grisea* (Magnaporthales) [23], and* Cladosporium fulvum* (Capnodiales) [20], HFBs are considered as virulence factors because they reduce exposure of pathogen-associated molecular patterns (PAMPs) and antigens to receptors of the immune system. This prevents the PAMPs from being recognized by the immune cells of plants and animals, including humans [15]. HFBs are also involved in symbiotic interactions, such as those between lichens and mycorrhizae [24, 25]. HFBs play a role in development and morphogenesis in the majority of the filamentous fungi and influence spore properties (for reviews, see [26–29]). Thus, we hypothesized that the respective genes could be suitable targets for ecological genetic investigation.

Molds from the common mycoparasitic and saprotrophic genus *Trichoderma* (Hypocreales) have the highest diversity of HFB-encoding genes in their genomes among Ascomycota [7, 30, 31]. Some species are airborne [32, 33] and form either single or oligosporic clumps of mitotic spores (conidia), while other species form conidiospores in wet or slimy heads [34, 35] that are thought to be suitable for dispersal by water or arthropods [34–38]. We selected two common morphologically identical (cryptic) species of the *Harzianum* Clade: *T. harzianum* and *T. guizhouense* [36, 39]. These species diverged approximately 5 Mya [7]. The genome of the reference strain for *T. harzianum* CBS 226.95 [40] contains eleven HFB-encoding genes, while that of *T. guizhouense* NJAU 4742 [40] contains nine such genes [7]. Although the preferential dispersal mode for each of these species is not defined, strains of the *Harzianum* Clade can form wet heads [34] or be air-borne [33, 37].

In this study, we tested the hypothesis that the HFB-related traits are essential factors for fungal fitness, and therefore, the analysis of such traits in closely related and cryptic *Trichoderma* species may reveal evolutionary forces that drive fungal speciation.

## Materials and methods

### Strains and culture conditions

Genome-sequenced strains *T. harzianum* CBS 226.95 (Th) [40] and *T*. *guizhouense* NJAU 4742 (Tg) [40] from the *Harzianum* Clade [7, 36] were used as the wild type and parental strains throughout the study. Additional strains used for determining *hfb* gene sequences, strain IDs in other collections, and the NCBI GenBank accession numbers of DNA barcode loci, and *hfb4* and *hfb10* sequences are listed in Supplementary information Table S1.

Fungal static liquid cultivation was performed with 50 mL flasks by inoculating 15 µL of 10^6^ spores µL^−1^ suspension to 15 mL of 30% Murashige Skoog basal salt mixture (Sigma-Aldrich, USA) supplemented with 1% glucose (MSG). Fungal cultures were incubated at 25 °C in darkness, and images were recorded by using the Canon EOS 70D (Canon, Japan). *Trichoderma* spores were collected from 7-day-old potato dextrose agar (PDA, Sigma-Aldrich, USA) cultures, and mycelia were filtrated out. All strains, unless otherwise specified, were maintained on PDA at 25 °C in darkness.

### Expression analysis of *hfb* genes and gene deletion

To study the expression of *hfb*s during *Trichoderma* development, fungal biomass corresponding to the three stages of the life cycle, namely, (i) the log-phase phase of growth in the substrate—48 h, (ii) the beginning of aerial hyphae formation - 72 h, and (iii) conidiation - 120 h, were collected from the cellophane-covered liquid cultures (6-cm Petri dishes) which were filled with 3 mL of MSG medium. Total RNA was extracted from each fungal biomass sample using the RNeasy Plant MiniKit (Qiagen, Germany) according the manufacturer’s protocol. cDNA was synthesized with the RevertAid™ First Strand cDNA Kit (Thermo Fischer Scientific, USA) using an oligo (dT)^18^ primer and calibrated by a NanoDrop spectrophotometer (Thermo Fischer Scientific, USA). qPCR was performed using qTOWER (Jena Analytics, Germany) for the genes of interest and calculated by the 2^-ΔΔCt^ method [41, 42] using the translation elongation factor 1 alpha (*tef1*) as the housekeeping gene [43, 44]. Amplification was performed using a total volume of 20 µL containing 10 µl of iQ SYBR Green Supermix (Bio-Rad, USA), 0.5 µM each primer and 5 ng µL^−1^ of cDNA. The program was set as follows: a thermal cycle for 6 min at 95 °C followed by 40 cycles of 30 s at 95 °C and 60 s at 60 °C, and a melting curve from 55 °C to 95 °C. All primers used in this study are given in Supplementary information Table S2.

Gene deletion mutants were obtained via gene replacement strategy using the poly-ethylene glycol (PEG)-mediated protoplast transformation procedure as described in Zhang et al. [44] with a hygromycin B cassette (*hph*, from the plasmid pPcdna1-hph, [45]) and/or a geneticin cassette (*neo*, from the plasmid pPki-Gen, [46]) (Supplementary information Fig. S1). Single- and double-deletion mutants were generated for the two most highly expressed genes *hfb4* and *hfb10* for each species, resulting in the following mutant library: _Th_Δ*hfb4*-3, _Th_Δ*hfb4*-11, _Th_Δ*hfb10*-2, _Th_Δ*hfb10*-17, _Th_Δ*hfb4*_Th_Δ*hfb10*-27, _Th_Δ*hfb4*_Th_Δ*hfb10*-30 for *T. harzianum*, and _Tg_Δ*hfb4*-1, _Tg_Δ*hfb4*-4, _Tg_Δ*hfb10*-2, _Tg_Δ*hfb10*-3, _Tg_Δ*hfb4*_Tg_Δ*hfb10*-2 and _Tg_Δ*hfb4*_Tg_Δ*hfb10*-11 for *T. guizhouense*.

### DNA extraction, PCR amplification and sequencing

For molecular phylogenetic analysis of *hfb* genes, fungal total DNA was extracted from the 48-h-old PDA cultures using the DNeasy Plant Mini Kit (Qiagen, Germany) according the manufacturer’s protocol. DNA fragments corresponding to *hfb4* or *hfb10* were respectively PCR-amplified with the primer pairs hfb4seq-F and hfb4seq-R, and hfb10seq-F, and hfb10seq-R (Supplementary information Table S2). The PCR reaction mixture (20 μL) contained 0.5 μM of each primer, 5 ng μL^−1^ of DNA, 10 μL of 2× Phanta Max Buffer mix and 2 U Phanta Max Super-Fidelity DNA Polymerase (Vazyme Biotech Co., Ltd, China). The thermal cycling process was set with the following program: 3 min at 95 °C for initial denaturing, 30 cycles of 15 s at 95 °C for denaturing, 15 s at 59 °C for annealing and 30 s at 72 °C, with a final extension at 72 °C for 5 min. Amplicons were sent for Sanger sequencing.

### Molecular evolutionary analyses of natural selection pressure

The *hfb* sequences and their corresponding amino acid sequences were retrieved either from the databases of the National Center for Biotechnology Information (NCBI) and the DOE Joint Genome Institute (JGI) or obtained by Sanger sequencing. Multiple sequence alignment was done using Muscle 3.8.31 [47] integrated in AliView 1.23 [48]. Maximum likelihood (ML) phylogenetic tree was constructed by using IQ-TREE 1.6.12 [49, 50] with the nucleotide substitution model selected based on the Bayesian Information Criterion, BIC, by ModelFinder [49] that integrated in IQ-TREE. Statistical support was inferred by 1000 bootstrapping replicates. ML analyses using EasyCodeML v1.21 program, a graphical interface for using CodeML [51, 52], were performed to determine the ratio (ω = *d*_N_/*d*_S_) of nonsynonymous/synonymous substitution rates, based on the *hfb* gene tree topologies constructed by the ML method. To evaluate variation in selective pressure between the lineages, CodeML branch models were applied under two *a priori* assumptions: a one-ratio model (M0) in which one ω value was assumed for the entire tree and a two-ratio model (M2) in which ω values were allowed to vary between the selected foreground branch and the background branch [52]. Here, the lineages of *T. guizhouense* and *T. harzianum* were used as the foreground branches, in turn. Besides, branch-site models (Model A), which allows ω to vary both among sites and among branches, were applied to determine the contribution of adaptive evolution of sites in these branches [52]. Positive selection was assigned if ω > 1. Purifying selection was assigned if 0 < ω < 1.

### Estimation of the fitness-related phenotypes

#### Determination of fungal growth by Biolog Phenotype MicroArrays

Growth was monitored using Biolog FF Microplates, which include 95 wells with each containing a different carbon source and one well with water (BIOLOG, Hayward, USA), as described in [53] with the following modifications. Spores were harvested from the 7-day-old PDA cultures and suspended in milli-Q water. Spore concentration was adjusted using a Biolog turbidity meter at O.D. 590 nm to 10^7^ spores mL^−1^. Ninety μL of the spore suspension was dispensed into each well. The assays were carried out with at least three replicates per each genotype. The values of O.D.750 nm were measured 12, 18, 24, 36, 48, 60, 72, 96, 120, 144, and 168 h post inoculation. The plates were incubated in darkness at 25 °C.

#### Estimation of spore production potential using the artificial intelligence algorithm

The ability to reproduce by asexual spores was estimated as the formation of aerial hyphae and conidiation intensity. First, it was recorded using the high-resolution images (5472 × 3648 pixels) of the Biolog FF Microplates obtained with the Canon EOS 70D (equipped with a Canon 100 μm macro lens) 72, 96, 120, 144, and 168 h post inoculation. Second, quantitative information regarding aerial hyphae formation and conidiation dynamics in each well (carbon source) were analyzed using an artificial intelligence algorithm. Specifically, the position of every well was automatically detected by searching for blobs with a large saturation in HSV color space [54]. All detected wells were then cropped and normalized to a fixed size of 256 × 256 pixels. To estimate which regions were covered by hyphae or conidia, a machine learning algorithm based on the U-Net [55] was used. The algorithm was trained on a dataset consisting of 1920 wells of the cultivated Biolog FF Microplates, and their corresponding ground-truth annotations were created by the operation staffs. When analyzing an image, the U-Net classified each pixel to determine if the pixel was covered by hyphae or conidia. Based on the classification results, the percentage of the area covered by hyphae or conidia was calculated for each well. Thus, the above algorithm was used to automatically quantify the aerial mycelia and conidia abundance (% coverage) on each carbon source at the different time points measured, and was labeled as the REproduction Potential Artificial INTellegence assay (REPAINT) applicable for conidiation.

#### Spore dispersal assays

We carried out an air dispersal assay to evaluate the role of HFBs in this trait. Fungal cultures were grown on PDA for 21 days. The areas with roughly equal conidia abundance were cropped into 1 × 1 cm^2^ plugs and placed under constant air flow (0.3 m s^−1^) through a 30-cm-long pipe (stainless steel, dia. 9 cm). Dispersed spores were trapped by a 9 cm PDA plate installed on the opposite end of the pipe, and colony forming unites (CFU) per plate were counted after incubation for 30 h. O.D. 600 nm of spore suspension, washed from the culture plugs from the same plate, was used to normalize the conidia abundance between the cultures. The set up for the assay is presented in Supplementary information Fig. S2a.

The water dispersal assay was performed as described by Whiteford and Spanu [20], with modifications. Specifically, the amount of spores that could be transported by water droplets was measured by releasing 200 μL of water (containing 0.05% Tween-80) along a 1 × 2 cm^2^ conidiating culture plug inclined at an angle of 60 ° (Supplementary information Fig. S2b). The water that rolled across the conidia plug was collected at the bottom of the fungal culture incline. Collected spores were counted using a hemocytometer and a Biolog turbidity meter (O.D. 590 nm). The conidia abundance between samples were normalized as described above.

#### Spore stress resistance assays

In fungi, the resilience of spores to abiotic stress factors is an important component of the ability to pass genes to the next generation (fitness). Therefore, we tested the spore resistance to adverse environmental factors such as drought, freezing temperatures, and UV radiation [12, 13, 56, 57].

For the measurement of spore resistance to freeze, 200 μL of spore suspension (10^8^ spores mL^−1^) was frozen in −80 °C for 12 h and lyophilized. The dried spores were re-suspended in 200 μL water, and 100 μL of the suspension was spread (with a standard 10-fold dilution plating method) on a 9 cm PDA plate supplemented by 0.5% Triton-X100. The CFUs were then calculated by quartette after 48 h of incubation at 25 °C.

For the measurement of spore resistance to drought, the spores were exposed to desiccation conditions. For this, 200 μL of spore suspension (10^8^ spores mL^−1^) was dried at 40 °C for 4 days. Subsequently, the standard 10-fold dilution plating was used. The results were adopted if supported by at least two dilution series, otherwise a third repeat with another spore concentration was performed.

For the measurement of spore resistance to UV radiation, 100 μL of spore suspension (10^3^ spores mL^−1^) was spread on a PDA plate supplemented by 0.5% Triton-X100. Then, plates were exposed to UV radiation (95 μw cm^2^, Longpro, China) for seven min. CFUs were counted per plate after 36 h of incubation at 25 °C.

### Spore surface properties and ultrastructure

To determine the link between the presence of HFBs and properties of the spore surface, we carried out the spore surface hydrophobicity assay. For this purpose, a drop of 10 μL distilled water was placed on the surface of a mature conidiating colony (0.5 × 1 cm^2^) using a Krüss EasyDrop DSA20E (Germany). The wettability of the spores was then expressed by the water contact angle (θ) as described in [58].

Micromorphology of the spore surface of fungal colonies (28-day old) was investigated using a cryo-scanning electron microscope (cryo-SEM, Quorum PP3010T integrated onto a Hitachi SU8010 FE-SEM, Japan).

### Homology modeling of HFB4

To determine the structure of HFB4 proteins from *T. harzianum* CBS 226.95 and *T. guizhouense* NJAU 4742, homology models were generated with MODELLER 9v15 [59] using the HFB2 structure from *T. reesei* (PDB ID: 2B97). Overall solvent-accessible surface area and hydrophobicity were estimated as the total SASA calculated by VMD with 1.4 Angstrom probe radius [60] or using Gravy calculator (http://www.gravy-calculator.de/).

### Statistical data analysis

The expression pattern of *hfb* genes at three stages of fungal development was illustrated by a heatmap that generated in R (version 3.6.1). Genes were clustered with the complete linkage Euclidean distance algorithm. The relative expression ratio (folds) of each *hfb* gene to the reference house-keeping gene *tef1* was indicated by the color intensity. To analyze the shift of dispersal mode due to *hfb* deletion, a complete linkage hierarchical clustering was also performed for the dispersal data in R. One-way analysis of variance (ANOVA) and Tukey HSD multiple comparison analyses were performed using STATISTICA 6 (StatSoft, USA) to test whether the removal of *hfb* gene(s) significantly affected the spore dispersal ability (*n* ≥ 6), surface hydrophobicity (*n* = 12), resistance to different abiotic stressors (*n* = 8), growth (Biolog Phenotype MicroArrays, *n* ≥ 288), and reproductive efficiency (REPAINT, production of aerial hypha and conidia abundance) (*n* ≥ 288). The significance threshold was set at *P* < 0.05. Results were demonstrated using box plots constructed in R package or as scatter plots. The generated data sets regarding Biolog Phenotype MicroArrays and REPAINT, were subjected to the principal component analysis (PCA) using ClustVis online tool (https://biit.cs.ut.ee/clustvis/, [61]) to test whether *hfb* deletion explains variation in phenotypic traits, within and between species.

## Results

**Fig. 1:**
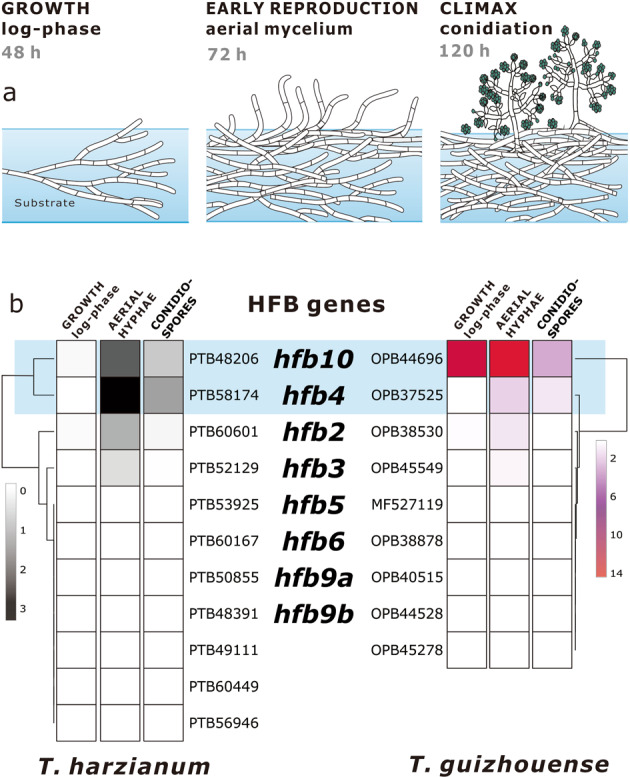
Expression of HFB-encoding genes during three stages of *Trichoderma* development. (**a**) Three stages of the asexual life cycle of *Trichoderma*. (**b**) A heatmap showing relative expression of HFB-encoding genes at each life stage in *T. harzianum* CBS 226.99 and *T. guizhouense* NJAU 4742, as quantified by qPCR in relation to the housekeeping gene *tef1*. The list of *hfb* genes is available from Kubicek et al. [7], and numbers indicate gene accessions in the NCBI GenBank.

To reveal HFBs that are associated with the sporulation of *T. harzianum* CBS 226.95 (Th) and *T. guizhouense* NJAU 4742 (Tg), we tested the expression of respective genes during the three stages of fungal development (Fig. 1a): (i) active growth (log-phase) shortly after germination when the mycelium is still developing in the substrate and no spores have formed (48 h), (ii) the formation of buoyant aerial mycelium shortly before conidiation (72 h), and (iii) mature conidiation during the climax of the life cycle (120 h) (Fig. 1b). The results showed that two genes were highly expressed during the formation of aerial mycelium and remained highly active during conidiation: _Th_PTB58174 / _Tg_OPB37525 (*hfb4*, [62]) and _Th_PTB48206/_Tg_OPB44696 (assigned as *hfb10* throughout this study, Supplementary information Table S1). Furthermore, *hfb2* and *hfb3* (Fig. 1b) showed low relative expression, while the expression of other genes was near the detection threshold level.

All *hfb*s showed low relative expression values during submerged growth except *hfb10* of Tg. *hfb4* and *hfb10* were relatively highly expressed during the reproduction of both species but in a different manner. Therefore, we produced a library of mutants lacking either one or both highly expressed *hfb* genes (*hfb4* and *hfb10*). Our library contained at least two mutant strains of each genotype per species (Supplementary information Table S1).

### Hydrophobins modulate the preferential dispersal mode of *Trichoderma*

**Fig. 2:**
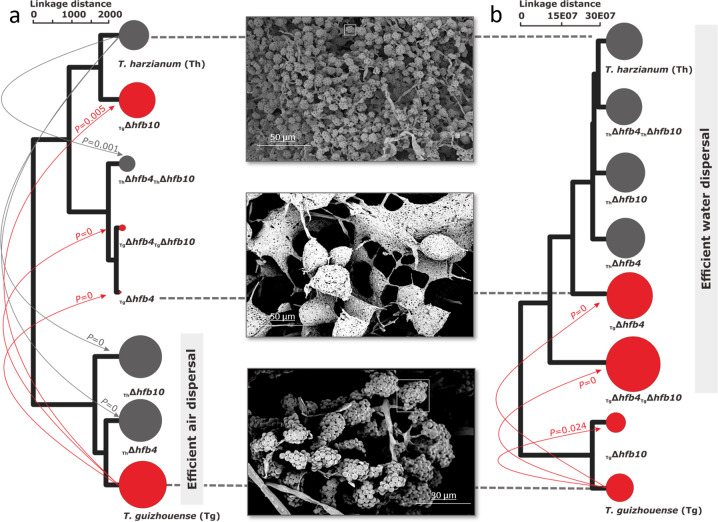
Switch in the preferential spore dispersal mode mediated by hydrophobins. Cluster analysis of the aerial (**a**) and water (**b**) dispersal efficiency measured for the wild type and *hfb*-mutant strains of the two *Trichoderma* species. Circle sizes indicate the relative number of transferred spores by one or another media. Arrows represent statistically significant shifts due to the deletion of one or two *hfb* genes (*P* < 0.05). Red and grey colors of circles and arrows highlight *T. guizhouense* and *T. harzianum*, respectively. Insets show the representative cryo-scanning electron microscope images (from 197 images) of mature spores for the three strains (dashed lines). Rectangles in the insets show spore clumps.

We first compared the two species for their affinity to aerial and water dispersal modes (see Materials and Methods and Supplementary information Fig. S1 for details). Tg spores were found to have about two-times higher affinity to air flow than Th (Supplementary information Fig. S2), while Th was more efficiently dispersed by water than Tg (Fig. 2). The deletion of *hfb4* resulted in an almost complete abolishment of air dispersal ability of the Tg spores, while deletion of *hfb10* showed a less pronounced loss of air dispersal capability, albeit still statistically significant (*P* = 0.005).

The double-deletion strains of Tg (_Tg_Δ*hfb4*_Tg_Δ*hfb10*) showed a similar phenotype to the _Tg_Δ*hfb4*. Thus, the deletion of *hfb4* severely impacted traits related to the aerial dispersal of Tg mutants. Interestingly, the single deletion of either *hfb4* or *hfb10* significantly improved the air dispersal ability of Th, making it equal to that of Tg (*P* = 0). However, double deletion strains of Th significantly (*P* = 0.001) reduced its abilities for air dispersal, similar to _Tg_Δ*hfb4* (Fig. 2 and Supplementary information Fig. S3). Cryo-SEM revealed that spores of the *hfb* mutant strains that were weaken in air dispersal were not aggregated in characteristic oligosporic conidial clumps present in these species. Instead, they were in large watery packs (Fig. 2) and appeared as a “dark spore phenotype” of the colony. The loss of *hfb* genes in Th did not change water dispersal (Fig. 2 and Supplementary information Fig. S3). Conversely, *hfb4* deletion or *hfb4* and *hfb10* deletion significantly (*P* = 0) increased the number of water-dispersed spores for Tg, while *hfb10* deletion resulted in a minor but significant improvement (*P* = 0.034).

The cluster analyses of air and water dispersal efficiencies (Fig. 2) of both species revealed that the deletion of *hfb4* in Tg possibly switched the primary dispersal medium from air to water. In Th, the deletion of any of these *hfb*s resulted in improved aerial dispersal but did not influence the dispersal by water. Taken together, these results allow us to conclude that HFB4 or HFB10 in Th prevent spores from being dispersed by air, and HFB4 in Tg prevents spores from being dispersed by water droplets. Such a strong difference in dispersal strategies was not expected for cryptic and closely related species.

### High spore surface hydrophobicity correlates with the preferential aerial dispersal mode of Tg

**Fig. 3:**
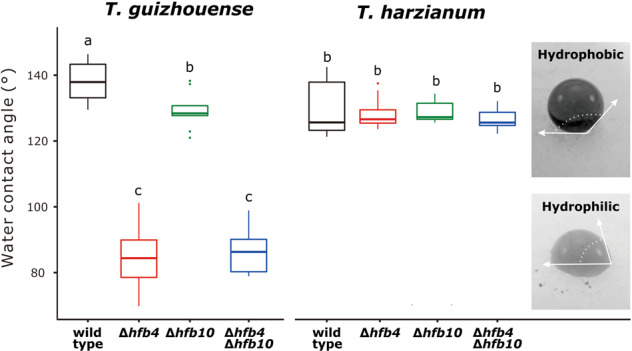
Spore surface hydrophobicity of *Trichoderma* wild type and HFB-mutant strains. Surface hydrophobicity of spores was estimated by the water contact angle of a water droplet applied on the fungal colony, as illustrated by the insets. Different letters represent statistically significant differences, *P* < 0.05. Spores with WCA < 90° were considered to be hydrophilic.

Aerial dispersal requires spore surface hydrophobicity, which is known to be provided by surface HFBs [63, 64]. Indeed, measurements of the water contact angle (WCA) showed that Tg spores were highly hydrophobic (Fig. 3), but _Tg_Δ*hfb4* and _Tg_Δ*hfb4*_Tg_Δ*hfb10* strains had hydrophilic spores (*P* = 0). Remarkably, _Tg_Δ*hfb10* strains had only minor but statistically significant declines (*P* = 0.01) in spore surface hydrophobicity, which would be considered negligible unless the efficiency of air dispersal of these strains is not changed.

Interestingly, our data show that even a minor reduction in surface hydrophobicity correlates with the reduced efficiency of aerial dispersal. The hydrophobicity of Th spores was slightly lower than that of Tg (*P* = 0.017) but equal to that of _Tg_Δ*hfb10* (Fig. 3). The absence of *hfb4* or *hfb10* in Th did not affect spore hydrophobicity. The analysis of spore surface by cryo-SEM did not reveal morphological changes associated with the species or tested genotypes (Supplementary information Fig. S4).

### Hydrophobins can change the resistance of spores to abiotic stress factors

**Fig. 4:**
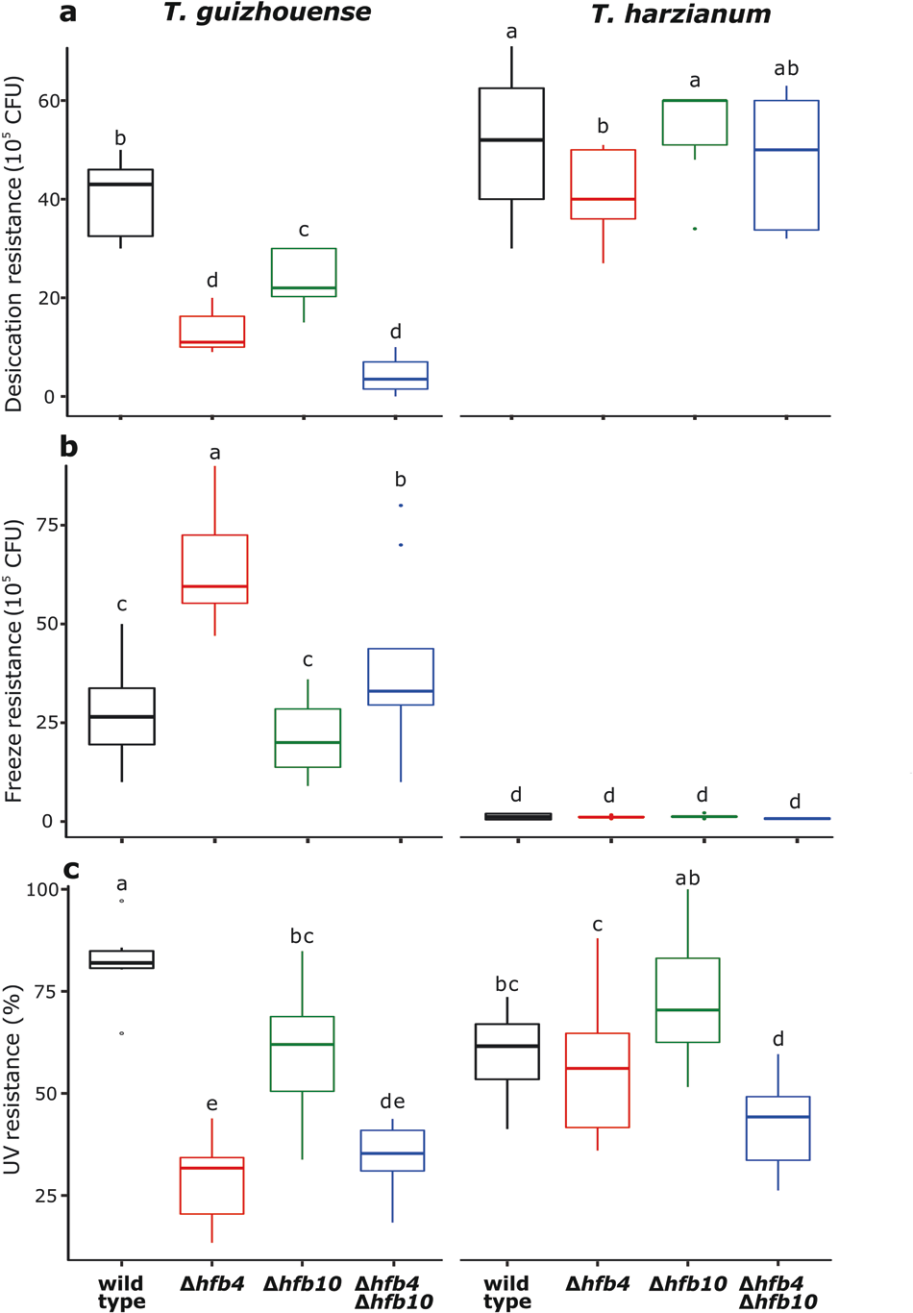
Stress resistance of spores against desiccation, freeze, and UV. The survival rate of spores for each strain was measured as CFU after spreading spores on PDA plates. UV resistance was given as the percentage of CFU comparison between the UV-treated and non-treated ones. Boxes with different letters represent a statistically significant difference from each other at the level of *P* < 0.05.

We tested whether HFB4 and HFB10 influence the resistance of spores to the most common abiotic stress factors by exposing spores to desiccation, freezing, and UV radiation (see Materials and Methods for the details). The results indicate that Tg strains exhibit significantly less desiccation resistance than Th (*P* < 0.05). The deletion of *hfb4* or *hfb10* significantly increased this vulnerability (*P* < 0.05, Fig. 4). The effect was stronger for *hfb4* than for *hfb10*. In Th, the same alteration was caused by the deletion of *hfb4*, but not *hfb10* (*P* > 0.05).

The UV resistance experiment revealed a similar trend for the mutants and species except that Tg spores were generally more UV-resistant. Tg spores also had considerably higher freeze resistance than Th (*P* = 0). Most surprisingly, the deletion of *hfb4* increased its freeze tolerance by more than twofold. The effect was reproduced in double-deletion strains and not present in _Tg_Δ*hfb10*. No such effect was noticed for Th (Fig. 4).

### Hydrophobins can strongly influence the growth and reproductive potential of *Trichoderma*

**Fig. 5:**
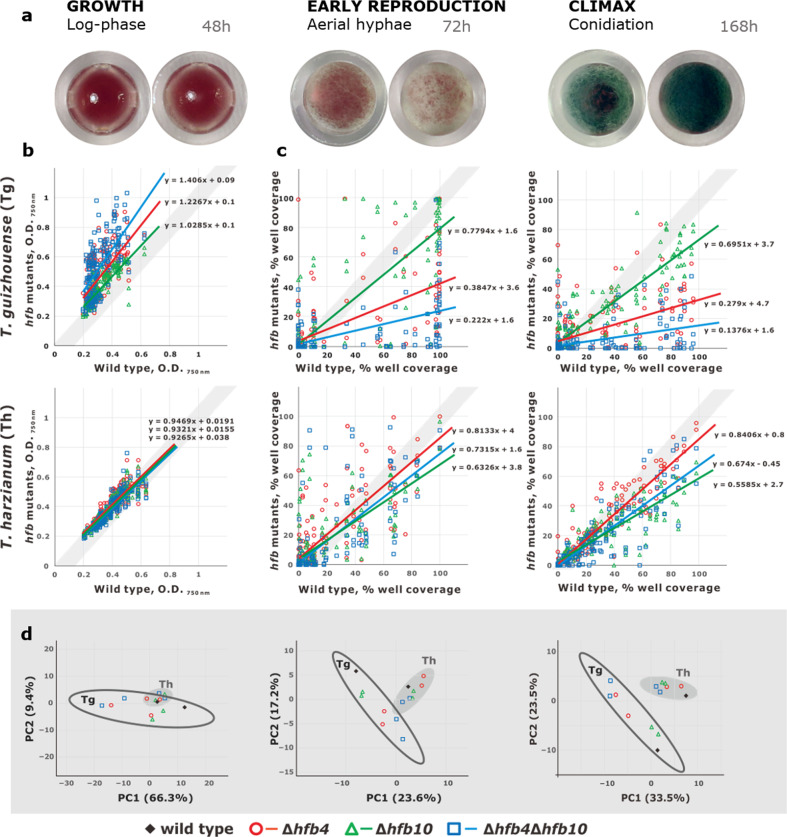
Impact of HFB4 and HFB10 on fungal growth and reproductive potential. (**a**) Three stages of the *Trichoderma* life cycle, which include the submerged growth, initial formation of aerial hyphae and conidiation. Insets show two representative cases for each stage imaged from the Biolog FF Microplates (well diameter: 6 mm). Scatter plots show the growth (**b**) measured as optical density at 750 nm (O.D.) using Biolog Phenotype Microarrays and the reproduction potential (**c**) estimated using the REproductive Potential Artificial INTelligence assay (REPAINT) of *hfb*-deficient mutants (Y-axis) compared to that of the wild-type strains (*X*-axis) on 95 carbon sources and on water. Each marker shows the value calculated on an individual carbon source. Two strains were analyzed for each deletion genotype. All data are presented in Supplementary information Figs. S5–S7, Dataset S1. The trend lines demonstrate the overall effect of *hfb* deletion on each mutant. Grey shadows on (**b**–**c**) correspond to the “no effect” zone. The principal component analysis (**d**) demonstrates the grouping pattern of the mutants and respective wild-type strains based on the data shown in (**b**) and (**c**). Corresponding time points for (**b**–**d**) are shown in (**a**).

Both *hfb* genes can influence the growth and reproduction of Tg. To avoid possible biases that are related to the medium composition, we investigated the impact of *hfb4* and *hfb10* genes on the vegetative growth, aerial hypha formation, and conidiation of Tg and Th (Fig. 5) by using Biolog Phenotype MicroArrays coupled with the REPAINT algorithm (see Materials and Methods and Supplementary information Figs. S5–S7 for details). Our results show that Tg mutants lacking *hfb4* or *hfb4* and *hfb10* had improved overall growth compared to the wild-type strain and the _Tg_Δ*hfb10* mutants (*P* = 0), whereas no difference was detected for Th strains (*P* > 0.05). However, improved growth on a certain carbon source did not guarantee a higher spore production potential since _Tg_Δ*hfb4* and _Tg_Δ*hfb4*_Tg_Δ*hfb10* strains showed significantly fewer aerial hyphae and lower conidia abundance than the wild-type strain (*P* = 0).

The removal of *hfb10* in Tg had a minor effect on the reproduction potential compared to those without *hfb4*, but, the double deletion of *hfb4* and *hfb10* in Tg resulted in a superposition effect (Fig. 5). No significant difference was found among the Th strains regarding vegetative growth and aerial hypha (*P* > 0.05). However, the conidial abundance of Th decreased significantly when *hfb10* was removed (*P* = 0). PCA (Fig. 5d) also supported the results that the deletion of HFBs has a more extensive influence on Tg than on Th and has a stronger effect on the fungal reproduction potential than the effect on vegetative growth. These assays suggest that *hfb4* can hinder fungal growth and development in some conditions as HFB4 affected the growth of Tg, as shown in Fig. 5b.

### HFB4 in *T. harzianum* evolves under strong positive selection pressure

The above analyses revealed that the two *hfb* genes are essential for at least several fitness-related traits in *Trichoderma*, but the impact of these proteins on predicted fitness was contradictory. Genes controlling multiple functions may experience multiple pressures of natural selection and evolve under the operation of the net effect of these forces [65, 66]. Therefore, we were interested in the mode of selection acting on each of these genes in each species.

For the analysis of selection pressure, we sequenced *hfb4* and *hfb10* genes for several additional strains available for Tg and Th (Supplementary information Figs. S8 and S9). As shown in Table 1, ω was estimated as 0.242 for HFB4 and 0.373 for HFB10 which showed the average ω overall sites in the protein and all lineages in the trees. These results indicate the dominating role of purifying (or stabilizing) selection in the evolution of the HFB proteins. However, model M2 assumes two different ω ratios between the branches and revealed that the value in HFB4 in the branch of the Th lineage (ω_1_ » 1) was significantly greater than the one of the background branches, indicating a strong positive selection that drives the evolution of *hfb4* in the Th species (Table 1). According to the branch-site model (Model A null vs. Model A), the sites 57 N (with posterior probability >95%) and 73D of HFB4 in Th were found to be evolving under positive selection pressure. In contrast, the value of ω was <1 when calculated for HFB4 in Tg and for HFB10 in Tg and Th lineages, indicating a primary purifying selection process for the proteins in the corresponding lineages.

**Table 1:** Log-likelihood values and parameter estimates for the natural selection pressure analysis of *hfb4* and *hfb10*.

		Model	np	Ln L	Estimates of parameters			Summary
*T. harzianum*	*hfb4*	M0 (one-ratio)	1	−1590.249	ω = 0.242			Positive selection (57N 95.2%, 73D 60.7%)
		M2 (two-ratio)	2	−1588.820	ω_0 _= 0.237	ω_1 _= 999.0*		
		Model A null (ω_2_ = 1)	3	−1517.869	—			
		Model A (ω_2_ > 1)	4	−1517.409	Site Class 0	*p*_0_ = 0.000	ω_0 _= 0.045	
					Site Class 1	*p*_1_ = 0.000	ω_1_ = 1	
					Site Class 2a	*p*_2_ + *p*_3_ = 1.000	ω_2 _= 999.0*	
					Site Class 2b			
	*hfb10*	M0 (one-ratio)	1	−1504.827	ω = 0.373	Purifying selection		Purifying selection
		M2 (two−ratio)	2	−1504.741	ω_0_ = 0.379	ω_1_ = 0.288		
		Model A null (ω_2_ = 1)	3	−1461.437	—			
		Model A (ω_2_ > 1)	4	−1461.437	Site Class 0	*p*_0_ = 0.304	ω_0_ = 0.039	
					Site Class 1	*p*_1_ = 0.203	ω_1_ = 1	
					Site Class 2a	p_2_ + *p*_3_ = 0.493	ω_2_ = 1.000	
					Site Class 2b			
*T. guizhouense*	*hfb4*	M0 (one-ratio)	1	−1590.249	ω=0.242	Purifying selection		Purifying selection
		M2 (two-ratio)	2	−1589.197	ω_0_=0.234	ω_1_ = 0.875		
		Model A null (ω_2_ = 1)	3	−1520.056	—			
		Model A (ω_2 _> 1)	4	−1520.056	Site Class 0	*p*_0_ = 0.675	ω_0_ = 0.046	
					Site Class 1	*p*_1 _= 0.325	ω_1 _= 1	
					Site Class 2a	*p*_2_ + *p*_3 _= 0.000	ω_2_ = 3.389	
					Site Class 2b			
	*hfb10*	M0 (one-ratio)	1	−1504.827	ω = 0.373			
		M2 (two-ratio)	2	−1504.200	ω_0_ = 0.390	ω_1_ = 0.201		
		Model A null (ω_2_ = 1)	3	−1464.461	—			
		Model A (ω_2_ > 1)	4	−1464.461	Site Class 0	*p*_0 _= 0.619	ω_0_ = 0.062	
					Site Class 1	*p*_1_ = 0.381	ω_1 _= 1	
					Site Class 2a	*p*_2_ + *p*_3 _= 0.000	ω_2 _= 1.000	
					Site Class 2b			

*np* number of parameters in ω distribution, *Ln L* Log-likelihood values. Positive selection sites shown in brackets are followed by their respective posterior probability. *ω = 999.0 is an arbitrary number reported by the software EasyCodeML v1.21 when the denominator (dS) is zero, indicating the situation that ω»1. Site numbering for HFB4 and HFB10 refer to the sequence of PTB58174 and PTB48206 from *T. harzianum* CBS 226.95, respectively.

The constraint of a strong positive selection pressure in the branch model on a protein-encoding gene is rarely detected [67, 68]. Thus, we used the advantage of *hfb4* being a core gene in *Trichoderma* spp. [7] and analyzed sequences of another 168 isolates that represent the major infrageneric clades of of the genus. This revealed that besides Th, *hfb4* also evolves under positive selection pressure in several other *Trichoderma* clades, such as *T. atroviride* and *T. aggressivum* species complexes. In other clades, it experiences a more commonly reported purifying selection pressure (Supplementary information Fig. S10). This later phenomenon was described previously for *Trichoderma hfb*s by Kubicek et al. [30]. Because *hfb10* is an orphan gene that is only found in the *Harzianum* Clade of *Trichoderma* [7], this type of analysis was not possible for this gene.

**Fig. 6:**
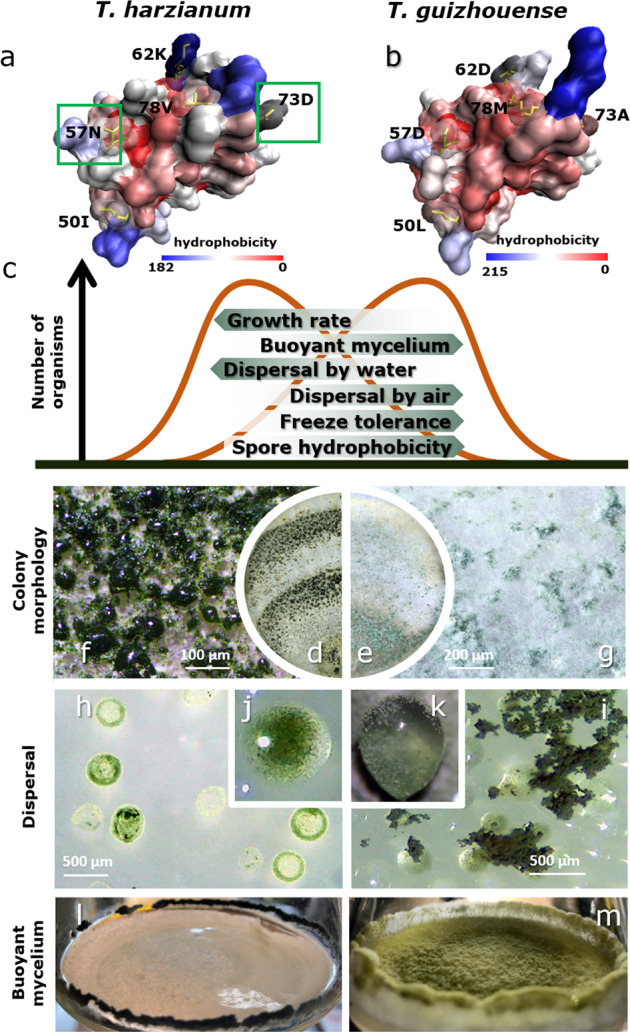
FB4 provides an evolutionary snapshot of the speciation in *Trichoderma*. H The homology models of HFB4 from *T. harzianum* (**a**) and *T. guizhouense* (**b**) were computed based on the 3D structure of HFB2 (PDB ID: 2B97) from *T. reesei*. Five of the six polymorphic sites in HFB4 (I50L, N57D, K62D, D73A and V78M) between Th and Tg, respectively, locate on the surface of the protein that may have an effect on the surface properties. The putative sites undergoing positive selection are framed. **c** Putative disruptive natural selection and directional shifts in HFB4-related features of *T. harzianum* and *T. guizhouense*. Colony morphologies associated with preferential water or aerial dispersal strategies are shown in (**d**) and (**f**) and (**e**) and (**g**), respectively. Naturally guttated drops containing spores of *T. harzianum* are shown in (**h**) and (**j**), while artificial water drops covered by air blown spores of *T. guizhouense* are shown in (**i**) and (**k**). The ability to form buoyant colonies is shown in (**l**) and (**m**) (diameter: 6 cm).

A remarkable finding of this study is the detection of the positive selection pressure operating on the *hfb4* gene in Th, even though the presence of this gene in this species is detrimental to some traits expected to be advantageous in our experiments. Interestingly, the homology modeling of HFB4 for each of the species against HFB2 of *T. reesei* and the analysis of its total hydrophobicity in silico revealed that they differ by six surface-located amino acids that make _Tg_HFB4 slightly more hydrophobic than _Th_HFB4 (Fig. 6 and Supplementary information Fig. S11).

## Discussion

In this study, we revealed that the alterations in a single HFB-encoding gene may cause changes that recapitulate the phenotypic differences thought to contribute to ecophysiological separation of species diverged at least 5 Mya [7]. *T. harzianum* sensu lato, the type species of the *Harzianum* species complex, is the most common taxon of the genus *Trichoderma* [7]. The first molecular phylogenetic studies pointed to considerable genetic polymorphism between morphologically indistinguishable *T. harzianum* strains [34, 36, 39, 69]. However, a broader multiloci analysis revealed traces of sexual recombination between genetically distant isolates and a continuum of physiological traits that point to a lack of consistent criteria that can be used for the reliable delineation of this species aggregate [39].

Later on, *T. harzianum* sensu lato was divided into more than a dozen phylogenetic species that largely corresponded to the polymorphism of one of the *Trichoderma* DNA barcoding markers, a fragment of the *tef1* gene, but also left the issue of species criteria open for further investigation. Thus, *T. harzianum* sensu stricto [36, 39] and *T. guizhouense* [36, 70] were formally recognized as putatively cosmopolitan cryptic species [34]. A subsequent comparative genomic study supported the genetic distance between these (and other) species [7, 40]. However, it did not address the evolutionary forces that led to the diversification, leaving species criteria to be exclusively DNA-based.

Here, we revealed that a set of ecophysiological fitness-related parameters describing development, dispersal, and stress resistance can reliably distinguish these species, making them non-cryptic. More surprisingly, most of these parameters were dependent on the presence of one or two HFB-encoding genes, at least in one of the species. Thus, the *hfb4* gene influences fitness-related traits that have species-specific differences in their manifestation. Figure 6c depicts a diagram of putative disruptive natural selection acting on fitness-related traits of *T. guizhouense* and *T. harzianum* and possibly resulting in the formation of these two species. The traits suggest that *T. harzianum* is likely to be a preferentially pluviophilous^1^ (dispersed by rain droplets), fast-growing fungus that rapidly produces spores when conditions become appropriate. Albeit more profound, a similar rapid life cycle is known for some coprophilic fungi such as *Podospora anserina* (Sordariales, Ascomycota), which evolved quick reproduction regimes due to the short-term availability of suitable substrate (dung) [71]. Interestingly, *P. anserina* is also one of a few senescent filamentous fungi with a fixed duration of the life cycle [72]. In our study, we also noted a faster and shorter life span of *T. harzianum* than *T. guizhouense* (Supplementary information Figs. S5–S7 and Dataset S1), which requires further investigation.

*T. guizhouense* forms a long-lasting buoyant mycelium (Fig. 6) that is suitable for the long-term production of stress-resistant anemophilous (air-dispersed) spores [22]. The sum of HFB4-related phenotypes of this species points to the similarity of *T. guizhouense* to the group of aero-aquatic (or amphibious) fungi [73, 74]. Such fungi can grow well in water and have adaptations for forming aerial reproductive mycelia. Usually, spores of aero-aquatic fungi such as *Helicodendron* spp. [75] have characteristic shapes or appendages that improve their ability to float on the surface of water. In the case of *T. guizhouense*, HFBs (mainly HFB4) can substitute these morphological structures because the appearance of this protein on the spore surface allows the fungus to reduce the surface tension of water and grow out of the liquid medium. This makes spores hydrophobic and air-dispersible and increases their resistance to low temperature and UV radiation.

The microscopic morphological features of *T. guizhouense* and *T. harzianum* are identical [36, 69], and macromorphologies of their young colonies are similar. Nevertheless, our results indicate that the mature colonies have significant differences that appear at the conidial stage. Thus, after 10–14 days of incubation at 25 °C, *T. guizhouense* remains fluffy and dry, while *T. harzianum* develops characteristic drops over conidiating areas (“dark spore phenotype”) that may be guttated by the fungus or come from condensed environmental moisture (Fig. 6). This correlates with the affinity of *T. harzianum* to form conidia in wet or slime heads that were described by Jaklitsch [34] and observed in this study. Many spores of common molds such as *Cladosporium* spp., *Penicillium* spp., *Alternaria* spp. (Pleosporales), and *Aspergillus* spp. are generated in dry chains and thus become airborne because this enables long-distance transfer [15]. Furthermore, falling spores will be exposed to a series of unfavorable environmental conditions, such as UV radiation or temperatures as low as −80 °C [12, 13, 56]. In agreement with this, spores of preferentially anemophilous *T. guizhouense* were resistant to freezing. Spores of *T. harzianum* did not survive low-temperature treatment. Therefore, our data suggest that this species (*T. harzianum*) cannot be dispersed over long distances by wind as it is linked to low temperatures. However, the species is cosmopolitan and is commonly found in soil all over the world [7, 36, 39]. Thus, the mechanism of its efficient dispersal may be associated with arthropods and remains to be discovered [37].

The deletion of *hfb4* in *T. guizhouense* resulted in a drastic increase of water dispersal, while the deletion of the same gene in *T. harzianum* resulted in increased anemophily. Remarkably, the colony morphologies associated with anemophilous dispersal strategies (Fig. 6) can be reversed to pluviophilous if *hfb4* is deleted in *T. guizhouense* (Supplementary information Fig. S12, [76]). This brought us to the comparison of HFB4 in the two species. The results of the calculated total surface hydrophobicity and the selection pressure analysis of the two HFB4s (*hfb4*s) suggest that positive selection at a fraction of sites contributes to the increased rate of amino acid substitution in HFB4 along the *T. harzianum* branch, while the functional constraint in HFB4 and in HFB10 purifies the mutation of these proteins along the *T. guizhouense* branch, as well as HFB10 in *T. harzianum*. Asexually reproducing lineages are expected to evolve relatively rapidly because they have a reduced effective population size, and their genes are more likely to experience selective sweeps that drive mutations to fixation [5, 77–79]. Therefore, as a clonal species [7, 39] that reproduces mitotically (asexually), *T. harzianum* evolves at faster rates than the sexually reproducing species. *T. guizhouense* is a putative holomorphic species that reproduces sexually and asexually [36], which also supports that its *hfb4* could evolve more slowly and that the operation of directional selection is prolonged, making it visible in more diverse habitats. Even though we have not defined the preferred habitats of *T. guizhouense* and *T. harzianum* in nature, it is reasonable to conclude they will be at least partially distinct and that the distinction has been arisen by positive selection for traits associated with HFB4.

The genes undergoing positive selection pressure reported so far are frequently found in groups involved in the regulation of reproduction and immunity [68] and mainly from the human genome and genomes of some plants. Only a few fungal genes have been shown to evolve under positive selection, such as the proline-rich antigen (*PRA*) gene in *Coccidioides* spp. [80] and the trichothecene mycotoxin gene in *Fusarium* spp. [81]. In our case, the strong positive selection on _Th_HFB4 strengthened the importance of reproduction-related genes involved in the speciation process, although HFBs do not control the reproductivity directly but can affect the fitness (including dispersal) of the progeny.

To verify our results, we tested the operation of the natural selection of HFB4 in a broad set of 170 *Trichoderma* strains (Supplementary information Fig. S10). This revealed that HFB4 evolves under the operation of positive selection in at least two other clades: one comprising strains of the polymorphic and common species *T. atroviride* and a clade consisting of *T. aggressivum*, *T. tawa*, *T. alni*, and *T. epimyces*. Interestingly, all these clades contain species with sexual and asexual life cycles. In summary, our results highlight the strong involvement of HFB4 and other hydrophobins in the species radiation of *Trichoderma* and related fungi that deserve future attention.

This study revealed that HFBs control multiple phenotypic and fitness-related traits. Although all features considered here could be involved in fitness, they may either be in conflict with one another or have a synergistic effect. This ultimately leads to the development of adaptive compromises that reflect the net effect of different selection pressures [82, 83]. Thus, the loss of HFB4 in *T. guizhouense* abolished the in vitro phenotype related to aerial dispersal, but the spores became more resistant to freezing temperatures. Together, this increases the chances for survival of these mutants if air remains the only medium for dispersal. Correspondingly, when the amount of water-dispersed spores increased because of the lack of HFB4 in *T. guizhouense*, the survival ratio in desiccation stress decreased. Similar compromising trait pairs can also be found in *T. harzianum*, whose spores would not survive low temperatures if anemophilous dispersal becomes preferential.

Phenotypic variation plays crucial roles in natural selection. However, it is difficult to integrate measurements of different fungal phenotypic traits in in vitro assessments of fitness. The measurements of fungal fitness have been restricted to development-related parameters, such as growth and the amount of spores [8]. In this study, we offer a toolkit that is based on eight parameters that can be further tested for fitness measurements to describe the development, dispersal, and stress resistance of spores. The novelty of our approach is the application of REPAINT assay to assessment of growth and sporulation which permitted the rapid integration of multiple traits across different carbon sources making high-throughput analyses become possible. Similar multiparametric phenotype assays may be customized for individual life cycles of fungi by adding specific parameters such as responses to illumination for fungi with circadian rhythms (*Neurospora crassa* [84]) or growth at temperatures of the human body for pathogens (dermatophytes or Aspergilli [85]).

We showed that HFBs were essential for phenotypes associated with spore dispersal and influence the resistance of spores to environmental stressors. Thus, multiple aspects of growth and spore dispersal are expected to be important to fungal ecology. Hence, comparison of HFBs between closely related and cryptic *Trichoderma* species may reflect ecological adaptations. For example, we can speculate that anemophilous dispersal and highly hydrophobic spores became advantageous in aquatic habitats, thus possibly forcing HFB4 of *T. guizhouense* towards a more hydrophobic state. Further investigations on the evolution of HFB4 surface activity and ecology of both species will reveal the vector of environmental adaptations in these fungi. Moreover, the application of customized assays of such fitness-related phenotypes as dispersal efficiencies, stress resilience, and competitive vigor in various habitats and microcosms may shed light on the evolutionary forces that shape species radiation in different fungi, as well as explain the genetic polymorphism in numerous complexes of cryptic species. Combined with advances in fungal genomics, multi-parametric fitness assays could bring fungi in focus for ecological genomic and ecological genetic investigations.

## Supplementary information

SUPPLEMENTAL MATERIAL

Dataset 1

## Footnotes

1 We avoid using a common term *hydrophilous* (dispersed by water) because spores of fungi dispersed by water streams, such as rivers or seas, usually sink to the bottom, germinate, and then form buoyant aerial mycelia producing spores that may be again dispersed by water or through air. *T. harzianum* has strongly reduced its ability to form aerial mycelium, and therefore, this species is not typically hydrophilous. However, our data point to the affinity of *T. harzianum* spores to water drops originating either from guttation or environmental moisture (rain, dew, splashes). Therefore, we use the term *pluviophilous*, which refers to such droplets.

## References

[1] Choi J, Kim SH (2017). A genome tree of life for the fungi kingdom. Proc Natl Acad Sci USA.

[2] Hawksworth DL, Lucking R. Fungal diversity revisited: 2.2 to 3.8 million species. Microbiol Spectr. 2017;5:FUNK-0052-2016. 10.1128/microbiolspec.funk-0052-2016 PMC11687528 28752818

[3] Hyde KD, Xu J, Rapior S, Jeewon R, Lumyong S, Niego AGT (2019). The amazing potential of fungi: 50 ways we can exploit fungi industrially. Fungal Div.

[4] Crous PW, Shivas RG, Quaedvlieg W, van der Bank M, Zhang Y, Summerell BA (2014). Fungal Planet description sheets: 214-280. Persoonia.

[5] Taylor JW, Jacobson DJ, Kroken S, Kasuga T, Geiser DM, Hibbett DS (2000). Phylogenetic species recognition and species concepts in fungi. Fungal Genet Biol.

[6] de Vries RP, Riley R, Wiebenga A, Aguilar-Osorio G, Amillis S, Uchima CA (2017). Comparative genomics reveals high biological diversity and specific adaptations in the industrially and medically important fungal genus *Aspergillus*. Genome Biol.

[7] Kubicek CP, Steindorff AS, Chenthamara K, Manganiello G, Henrissat B, Zhang J (2019). Evolution and comparative genomics of the most common *Trichoderma* species. BMC Genomics.

[8] Pringle A, Taylor J (2002). The fitness of filamentous fungi. Trends Microbiol.

[9] Gilchrist MA, Sulsky DL, Pringle A (2006). Identifying fitness and optimal life-history strategies for an asexual filamentous fungus. Evolution.

[10] Golan JJ, Pringle A. Long-distance dispersal of fungi. Microbiol Spectr. 2017;5:FUNK-0047-2016. 10.1128/microbiolspec.funk-0047-2016 PMC11687522 28710849

[11] Wyatt TT, Wosten HA, Dijksterhuis J (2013). Fungal spores for dispersion in space and time. Adv Appl Microbiol.

[12] Fuller KK, Ringelberg CS, Loros JJ, Dunlap JC. The fungal pathogen *Aspergillus fumigatus* regulates growth, metabolism, and stress resistance in response to light. mBio. 2013;4. 10.1128/mBio.00142-13 PMC3604765 23532976

[13] Norros V, Karhu E, Norden J, Vahatalo AV, Ovaskainen O (2015). Spore sensitivity to sunlight and freezing can restrict dispersal in wood-decay fungi. Ecol Evol.

[14] Roper M, Seminara A, Bandi MM, Cobb A, Dillard HR, Pringle A (2010). Dispersal of fungal spores on a cooperatively generated wind. Proc Natl Acad Sci USA.

[15] Aimanianda V, Bayry J, Bozza S, Kniemeyer O, Perruccio K, Elluru SR (2009). Surface hydrophobin prevents immune recognition of airborne fungal spores. Nature.

[16] Wösten HA (2001). Hydrophobins: multipurpose proteins. Annu Rev Microbiol.

[17] Bayry J, Aimanianda V, Guijarro JI, Sunde M, Latge JP (2012). Hydrophobins–unique fungal proteins. PLoS Pathog.

[18] Aimanianda V, Latge JP (2010). Fungal hydrophobins form a sheath preventing immune recognition of airborne conidia. Virulence.

[19] Zhang S, Xia YX, Kim B, Keyhani NO (2011). Two hydrophobins are involved in fungal spore coat rodlet layer assembly and each play distinct roles in surface interactions, development and pathogenesis in the entomopathogenic fungus, *Beauveria bassiana*. Mol Microbiol.

[20] Whiteford JR, Spanu PD (2001). The hydrophobin HCf-1 of *Cladosporium fulvum* is required for efficient water-mediated dispersal of conidia. Fungal Genet Biol.

[21] Guzman-Guzman P, Aleman-Duarte MI, Delaye L, Herrera-Estrella A, Olmedo-Monfil V (2017). Identification of effector-like proteins in *Trichoderma* spp. and role of a hydrophobin in the plant-fungus interaction and mycoparasitism. BMC Genet.

[22] Lugones LG, de Jong JF, de Vries OM, Jalving R, Dijksterhuis J, Wosten HA (2004). The SC15 protein of *Schizophyllum commune* mediates formation of aerial hyphae and attachment in the absence of the SC3 hydrophobin. Mol Microbiol.

[23] Beckerman JL, Ebbole DJ (1996). *MPG1*, a gene encoding a fungal hydrophobin of *Magnaporthe grisea*, is involved in surface recognition. Mol Plant Microbe.

[24] Rineau F, Lmalem H, Ahren D, Shah F, Johansson T, Coninx L (2017). Comparative genomics and expression levels of hydrophobins from eight mycorrhizal genomes. Mycorrhiza.

[25] Scherrer S, Haisch A, Honegger R (2002). Characterization and expression of *XPH1*, the hydrophobin gene of the lichen-forming ascomycete *Xanthoria parietina*. N Phytologist.

[26] Bailey MJ, Askolin S, Horhammer N, Tenkanen M, Linder M, Penttila M (2002). Process technological effects of deletion and amplification of hydrophobins I and II in transformants of *Trichoderma reesei*. Appl Microbiol Biotechnol.

[27] Fuchs U, Czymmek KJ, Sweigard JA (2004). Five hydrophobin genes in *Fusarium verticillioides* include two required for microconidial chain formation. Fungal Genet Biol.

[28] Winefield RD, Hilario E, Beever RE, Haverkamp RG, Templeton MD (2007). Hydrophobin genes and their expression in conidial and aconidial *Neurospora species*. Fungal Genet Biol.

[29] Sevim A, Donzelli BG, Wu D, Demirbag Z, Gibson DM, Turgeon BG (2012). Hydrophobin genes of the entomopathogenic fungus, *Metarhizium brunneum*, are differentially expressed and corresponding mutants are decreased in virulence. Curr Genet.

[30] Kubicek CP, Baker S, Gamauf C, Kenerley CM, Druzhinina IS (2008). Purifying selection and birth-and-death evolution in the class II hydrophobin gene families of the ascomycete *Trichoderma/Hypocrea*. BMC Evol Biol.

[31] Seidl-Seiboth V, Gruber S, Sezerman U, Schwecke T, Albayrak A, Neuhof T (2011). Novel hydrophobins from *Trichoderma* define a new hydrophobin subclass: protein properties, evolution, regulation and processing. J Mol Evol.

[32] Kim JY, Kwon HW, Lee DH, Ko HK, Kim SH (2019). Isolation and characterization of airborne mushroom damaging *Trichoderma* spp. from indoor air of cultivation houses used for Oak wood mushroom production using sawdust media. Plant Pathol J.

[33] Rao CY, Riggs MA, Chew GL, Muilenberg ML, Thorne PS, Van Sickle D (2007). Characterization of airborne molds, endotoxins, and glucans in homes in New Orleans after Hurricanes Katrina and Rita. Appl Environ Microbiol.

[34] Jaklitsch WM (2009). European species of *Hypocrea* Part I. The green-spored species. Stud Mycol.

[35] Jaklitsch WM (2011). European species of *Hypocrea* part II: species with hyaline ascospores. Fungal Divers.

[36] Chaverri P, Branco-Rocha F, Jaklitsch W, Gazis R, Degenkolb T, Samuels GJ (2015). Systematics of the *Trichoderma harzianum* species complex and the re-identification of commercial biocontrol strains. Mycologia.

[37] Atriztan-Hernandez K, Moreno-Pedraza A, Winkler R, Markow T, Herrera-Estrella A. *Trichoderma atroviride* from predator to prey: role of the mitogen-activated protein kinase *tmk3* in fungal chemical defense against fungivory by *Drosophila melanogaster* larvae. Appl Environ Microbiol. 2019;85:e01825–18. 10.1128/AEM.01825-18 PMC6328759 30389761

[38] Yamaguchi K, Tsurumi Y, Suzuki R, Chuaseeharonnachai C, Sri-Indrasutdhi V, Boonyuen N (2012). *Trichoderma matsushimae* and *T. aeroaquaticum*: two aero-aquatic species with *Pseudaegerita*-like propagules. Mycologia.

[39] Druzhinina IS, Kubicek CP, Komon-Zelazowska M, Mulaw TB, Bissett J. The *Trichoderma harzianum* demon: complex speciation history resulting in coexistence of hypothetical biological species, recent agamospecies and numerous relict lineages. BMC Evolut Biol. 2010;10:94. 10.1186/1471-2148-10-94 PMC2858147 20359347

[40] Druzhinina IS, Chenthamara K, Zhang J, Atanasova L, Yang DQ, Miao YZ, et al. Massive lateral transfer of genes encoding plant cell wall-degrading enzymes to the mycoparasitic fungus *Trichoderma* from its plant-associated hosts. Plos Genetics. 2018;14:e1007322. 10.1371/journal.pgen.1007322 PMC5908196 29630596

[41] Alcazar-Fuoli L, Clavaud C, Lamarre C, Aimanianda V, Seidl-Seiboth V, Mellado E (2011). Functional analysis of the fungal/plant class chitinase family in *Aspergillus fumigatus*. Fungal Genet Biol.

[42] Schmittgen TD, Livak KJ (2008). Analyzing real-time PCR data by the comparative CT method. Nat Protoc.

[43] Zhang J, Bayram Akcapinar G, Atanasova L, Rahimi MJ, Przylucka A, Yang D (2016). The neutral metallopeptidase NMP1 of *Trichoderma guizhouense* is required for mycotrophy and self-defence. Environ Microbiol.

[44] Zhang J, Miao Y, Rahimi MJ, Zhu H, Steindorff A, Schiessler S (2019). Guttation capsules containing hydrogen peroxide: an evolutionarily conserved NADPH oxidase gains a role in wars between related fungi. Environ Microbiol.

[45] Uzbas F, Sezerman U, Hartl L, Kubicek CP, Seiboth B (2012). A homologous production system for *Trichoderma reesei* secreted proteins in a cellulase-free background. Appl Microbiol Biotechnol.

[46] Seiboth B, Karimi RA, Phatale PA, Linke R, Hartl L, Sauer DG (2012). The putative protein methyltransferase LAE1 controls cellulase gene expression in *Trichoderma reesei*. Mol Microbiol.

[47] Edgar RC (2004). MUSCLE: a multiple sequence alignment method with reduced time and space complexity. BMC Bioinform.

[48] Larsson A (2014). AliView: a fast and lightweight alignment viewer and editor for large datasets. Bioinformatics.

[49] Kalyaanamoorthy S, Minh BQ, Wong TKF, von Haeseler A, Jermiin LS (2017). ModelFinder: fast model selection for accurate phylogenetic estimates. Nat Methods.

[50] Nguyen LT, Schmidt HA, von Haeseler A, Minh BQ (2015). IQ-TREE: a fast and effective stochastic algorithm for estimating maximum-likelihood phylogenies. Mol Biol Evol.

[51] Gao FL, Chen CJ, Arab DA, Du ZG, He YH, Ho SYW (2019). EasyCodeML: a visual tool for analysis of selection using CodeML. Ecol Evol.

[52] Yang Z (2007). PAML 4: Phylogenetic analysis by maximum likelihood. Mol Biol Evol.

[53] Druzhinina IS, Schmoll M, Seiboth B, Kubicek CP (2006). Global carbon utilization profiles of wild-type, mutant, and transformant strains of *Hypocrea jecorina*. Appl Environ Microbiol.

[54] Smith AR (1978). Color gamut transform pairs. ACM SIGGRAPH. Comput Graph.

[55] Ronneberger O, Fischer P, Brox T. U-Net: convolutional networks for biomedical image segmentation. In: Navab N, Hornegger J, Wells W, Frangi A (eds). *Medical Image Computing and Computer-Assisted Intervention – MICCAI 2015*. Springer, Cham, 2015, pp. 234–41.

[56] Harrison JG, Lowe R (1987). Wind dispersal of conidia of *Botrytis* spp. pathogenic to *Vicia faba*. Plant Pathol.

[57] Nagarajan S, Singh DV. Long-distance dispersion rust pathogens. Annu Rev Phytopathol. 1990;28:139–53. 10.1146/annurev.py.28.090190.001035 20540608

[58] Przylucka A, Akcapinar GB, Bonazza K, Mello-de-Sousa TM, Mach-Aigner AR, Lobanov V (2017). Comparative physiochemical analysis of hydrophobins produced in *Escherichia coli* and *Pichia pastoris*. Colloids Surf B Biointerfaces.

[59] Webb B, Sali A (2016). Comparative protein structure modeling using. Modeller.

[60] Humphrey W, Dalke A, Schulten K (1996). VMD: visual molecular dynamics. J Mol Graph.

[61] Metsalu T, Vilo J (2015). ClustVis: a web tool for visualizing clustering of multivariate data using principal component analysis and heatmap. Nucleic Acids Res.

[62] Espino-Rammer L, Ribitsch D, Przylucka A, Marold A, Greimel KJ, Herrero Acero E (2013). Two novel class II hydrophobins from *Trichoderma* spp. stimulate enzymatic hydrolysis of poly(ethylene terephthalate) when expressed as fusion proteins. Appl Environ Microbiol.

[63] Grunbacher A, Throm T, Seidel C, Gutt B, Rohrig J, Strunk T (2014). Six hydrophobins are involved in hydrophobin rodlet formation in *Aspergillus nidulans* and contribute to hydrophobicity of the spore surface. PLoS ONE.

[64] Whiteford JR, Lacroix H, Talbot NJ, Spanu PD (2004). Stage-specific cellular localisation of two hydrophobins during plant infection by the pathogenic fungus *Cladosporium fulvum*. Fungal Genet Biol.

[65] Stuefer JF, Van Hulzen JB, During HJ (2002). A genotypic trade-off between the number and size of clonal offspring in the stoloniferous herb *Potentilla reptans*. J Evol Biol.

[66] Wolf JB, Brodie Iii ED, Cheverud JM, Moore AJ, Wade MJ (1998). Evolutionary consequences of indirect genetic effects. Trends Ecol Evol.

[67] Goldman N, Yang Z (2008). Introduction. Statistical and computational challenges in molecular phylogenetics and evolution. Philos Trans R Soc Lond B Biol Sci.

[68] Yang ZH (ed). Computational molecular evolution, 1st edn. Oxford University Press: New York, USA, 2006.

[69] Chaverri P, Samuels GJ. *Hypocrea/Trichoderma* (Ascomycota, Hypocreales, Hypocreaceae): species with green ascospores. Stud Mycol. 2004;48:1–116.

[70] Li QR, Tan P, Jiang YL, Hyde KD, Mckenzie EHC, Bahkali AH (2013). A novel *Trichoderma* species isolated from soil in Guizhou, *T. guizhouense*. Mycol Prog.

[71] Gagny B, Rossignol M, Silar P (1997). Cloning, sequencing, and transgenic expression of *Podospora curvicolla* and *Sordaria macrospora* eEF1A genes: Relationship between cytosolic translation and longevity in filamentous fungi. Fungal Genet Biol.

[72] Geydan TD, Debets AJ, Verkley GJ, van Diepeningen AD (2012). Correlated evolution of senescence and ephemeral substrate use in the Sordariomycetes. Mol Ecol.

[73] Prokhorov VP, Bodyagin VV (2007). The ecology of aero-aquatic hyphomycetes. Moscow Univ Biol Sci Bull..

[74] Shearer CA, Descals E, Kohlmeyer B, Kohlmeyer J, Marvanová L, Padgett D (2007). Fungal biodiversity in aquatic habitats. Biodivers Conserv.

[75] Voglmayr H (1997). Two new aero-aquatic species of the hyphomycete genus *Helicodendron* from Austria. Plant Syst Evol.

[76] Ashkenazy H, Abadi S, Martz E, Chay O, Mayrose I, Pupko T (2016). ConSurf 2016: an improved methodology to estimate and visualize evolutionary conservation in macromolecules. Nucleic Acids Res.

[77] Fisher MC, Koenig GL, White TJ, Taylor JW (2000). Pathogenic clones versus environmentally driven population increase: analysis of an epidemic of the human fungal pathogen *Coccidioides immitis*. J Clin Microbiol.

[78] Huang JP, Leavitt SD, Lumbsch HT (2018). Testing the impact of effective population size on speciation rates - a negative correlation or lack thereof in lichenized fungi. Sci Rep.

[79] Taylor JW. Evolutionary perspectives on human fungal pathogens. Cold Spring Harb Perspect Med. 2014;5. 10.1101/cshperspect.a019588 PMC4561393 25384770

[80] Johannesson H, Vidal P, Guarro J, Herr RA, Cole GT, Taylor JW (2004). Positive directional selection in the proline-rich antigen (*PRA*) gene among the human pathogenic fungi *Coccidioides immitis*, *C. posadasii* and their closest relatives. Mol Biol Evol.

[81] Ward TJ, Bielawski JP, Kistler HC, Sullivan E, O’Donnell K (2002). Ancestral polymorphism and adaptive evolution in the trichothecene mycotoxin gene cluster of phytopathogenic *Fusarium*. Proc Natl Acad Sci USA.

[82] Rieseberg LH, Widmer A, Arntz AM, Burke JM (2002). Directional selection is the primary cause of phenotypic diversification. Proc Natl Acad Sci USA.

[83] Vaknin Y, Gan-Mor S, Bechar A, Ronen B, Eisikowitch D (2001). Are flowers morphologically adapted to take advantage of electrostatic forces in pollination?. N Phytologist.

[84] Liu Y, Bell-Pedersen D (2006). Circadian rhythms in *Neurospora crassa* and other filamentous fungi. Eukaryot Cell.

[85] McCormick A, Loeffler J, Ebel F. *Aspergillus fumigatus*: contours of an opportunistic human pathogen. Cell Microbiol. 2010;12:1535–43. 10.1111/j.1462-5822.2010.01517.x 20716206

