# Supplementary material for: Evolutionary compromises in fungal fitness: hydrophobins can hinder the adverse dispersal of conidiospores and challenge their survival
Source: ISME J. 2020 Jul 6;14(10):2610–24. doi: 10.1038/s41396-020-0709-0 (PMC7490268; doi:10.1038/s41396-020-0709-0)
Supplement: Supplementary file 1 — SUPPLEMENTAL MATERIAL [file 41396_2020_709_MOESM1_ESM.pdf]

Supplementary Information for *Cai et al.*

Evolutionary compromises in fungal fitness: *hydrophobins can hinder the adverse dispersal of conidiospores and challenge their survival*

Contents

Table S1. Strains used in this study. 2

Table S2. Primers used in this study. 3

Fig. S1. Mutant construction and verification. 6

Fig. S2. Schematic diagram of experimental design for air (a) and water (b) dispersal tests. 7

Fig. S3. Comparison of the ability of spores able to be dispersed by air or water between strains. 8

Fig. S4. Micromorphology of spores. 9

Fig. S5. The role of HFBs in regulating growth of *T. guizhouense* (a) and *T. harzianum* (b). 13

Fig. S6. The role of HFBs in regulating aerial hypha formation of *T. guizhouense* (a) and *T. harzianum* (b). 17

Fig. S7. The role of HFBs in regulating conidiation of *T. guizhouense* (a) and *T. harzianum* (b). 21

Fig. S8. Maximum likelihood phylogenetic trees of *hfb4* (a) and *hfb10* (b) genes from *T. guizhouense*, *T. harzianum* and their closely-related species. 22

Fig. S9. Codon sequence alignment of *hfb4* (a) and *hfb10* (b) from *T. guizhouense*, *T. harzianum* and their closely-related species. 24

Fig. S10. Maximum likelihood phylogram of the *hfb4* gene from 170 strains belonging to the *Trichoderma* genus. 25

Fig. S11. Homology modelling (a) and protein sequence alignment (b) of HFB4 from *T. guizhouense* NJAU 4742 and *T. harzianum* CBS 226.95. 26

Fig. S12. Impact of the HFB4-encoding gene in *T. harzianum* and *T. guizhouense* on fitness-related parameters and putative preferential dispersal modes of these fungi. 27

Dataset S1. (separate file). Raw data regarding fungal growth and reproduction potential. 28

References 28

Table S1. Strains used in this study.

Species	Strain ID	Description	NCBI Accession Number of nucleotide sequences		
			tef1	*hfb4*	*hfb10*
T. guizhouense	NJAU 4742	wild type	KP292611	OPB37525	OPB44696
	$_{\mathrm{Tg}}\Delta$*hfb4*-1	*hfb4* deleted mutant			
	$_{\mathrm{Tg}}\Delta$*hfb4*-4				
	$_{\mathrm{Tg}}\Delta$*hfb10*-2	*hfb10* deleted mutant		n.a.	
	$_{\mathrm{Tg}}\Delta$*hfb10*-3				
	$_{\mathrm{Tg}}\Delta$*hfb4*$_{\mathrm{Tg}}\Delta$*hfb10*-2	*hfb4* and *hfb10* deleted mutant			
	$_{\mathrm{Tg}}\Delta$*hfb4*$_{\mathrm{Tg}}\Delta$*hfb10*-11				
	TUCIM 53, PPRI 3909	wild type	EF113551	MN109965	MK579845
	TUCIM 1044, DAOM 231412		AY605764	MN109966	MK579843
	TUCIM 3009		FJ619249	MN109963	MN109968
	TUCIM 3611		JQ425711	MN109964	MK579844
T. harzianum	CBS 226.95^{T}	wild type, ex-type	AF348101	PTB58174	PTB48206
	$_{\mathrm{Th}}\Delta$*hfb4*-3	*hfb4* deleted mutant			
	$_{\mathrm{Th}}\Delta$*hfb4*-11				
	$_{\mathrm{Th}}\Delta$*hfb10*-2	*hfb10* deleted mutant		n.a.	
	$_{\mathrm{Th}}\Delta$*hfb10*-17				
	$_{\mathrm{Th}}\Delta$*hfb4*$_{\mathrm{Th}}\Delta$*hfb10*-27	*hfb4* and *hfb10* double deleted mutant			
	$_{\mathrm{Th}}\Delta$*hfb4*$_{\mathrm{Th}}\Delta$*hfb10*-30				
	TUCIM 217	wild type	AY605829	MK579846	MK579841
	TUCIM 1818		EF116558	MK579847	MK579842
T. afroharzianum	TUCIM 4803	wild type	MF179610	MN109967	MN515399
T. cf. *guizhouense*	TUCIM 1651		-	-	MN515401
T. lixii	TUCIM 2784, CBS 110080		FJ716622	-	MN515400

T, type strain.

TUCIM, TU Collection of Industrial Microorganisms, Vienna, Austria.

Table S2. Primers used in this study.

Primers	Sequences (5'-3')	Comments
4742h4-upF	TACGTTACAAGACTATCCCA	Deletion of *hfb4* in *T. guizhouense* NJAU 4742
4742h4-upR	CATATTGATGTAAGGTAGCTCTCGGATCCCTTGAACAATGTTTATTGCG	
4742h4-dnF	TATTCCATCTAAGCCATAGTACCCTCGAGATTCTCGATTAGCCGGTTGC	
4742h4-dnR	GAGTGGCTGCTATCTATCTA	
4742h4hph-F	GGATCCGAGAGCTACCTTACAT	
4742h4hph-R	CTCGAGGGTACTATGGCTTAGAT	
4742h4-F2	CAGGCGGTGATGGAGCAAA	Verification of *hfb4* deletion in *T. guizhouense* NJAU 4742
4742h4-R2 (hph158 R)	CAAGTACAACCTAACAGCTGAGCAC	
4742h4-F3	GAAGTTCTCTGCCATCGCTCTCT	
4742h4-R3	AGGTCGGCCTGAAGACTAGTTAGTA	
4742h4-F4 (hph430 F)	AGAAGGGCGTCGAGCATTGT	
4742h4-R4	ATTGGCACGAAGACCAGACATC	
4742h10-upF	CGTCTCAGTCTAGCTATAAT	Deletion of *hfb410* in *T. guizhouense* NJAU 4742
4742h10-upR	CATATTGATGTAAGGTAGCTCTCGGATCCGAGAATTGAAGACTAAGGAG	
4742h10-dnF	TATTCCATCTAAGCCATAGTACCCTCGAGGCGTACGAAGGTCCTTTGAT	
4742h10-dnR	AGCTCTTCAGGCCCTGGTGG	
4742h10hph-F	refer to 4742h4hph-F	
4742h10hph-R	refer to 4742h4hph-R	
4742h10-F2	CCTAAGGCTGTAAGCGAAAGAC	Verification of *hfb10* deletion in *T. guizhouense* NJAU 4742
4742h10-R2	refer to 4742h4-R2 (hph158 R)	
4742h10-F3	CCGTCTTCTTCACTGCTGTCCT	
4742h10-R3	CACCTTGGCAAAGGAGGTCTT	
4742h10-F4	refer to 4742h4-F4 (hph430 F)	
4742h10-R4	TAGGTGGCATGTTTAGCAATTTTCT	
4742h10neo-upF	refer to 4742h10-upF	Deletion of *hfb10* in $_{Tg}\Delta$*hfb4*-4 mutant
4742h10neo-upR	CTCGTATGACCTGAAGGAAATCAAAGCCGGAGAATTGAAGACTAAGGAG	
4742h10neo-dnF	CGCGACTATATATTTGTCTCTAATTGTACGCGTACGAAGGTCCTTTGAT	
4742h10neo-dnR	refer to 4742h10-dnR	
4742h10neo-F	CGGCTTTGATTTCCTTCAGG	
4742h10neo-R	GTACAATTAGAGACAAATATATAGTCGCGT	
4742h10neo-F2	refer to 4742h10-F2	Verification of *hfb10* deletion in $_{Tg}\Delta$*hfb4*-4 mutant
4742h10neo-R2 (neo500 R)	CCCAAAAAGTGCTCCTTCAATA	
4742h10neo-F3	refer to 4742h10-F3	
4742h10neo-R3	refer to 4742h10-R3	
4742h10neo-F4 (neo372 F)	ATATAAGATCGTTGGTGTCGATGTC	
4742h10neo-R4	refer to 4742h10-R4	
916h4-upF	TGTGTGAACACGTTACAAGACTATCC	Deletion of *hfb4* in *T. harzianum* CBS 226.95
916h4-upR	CATATTGATGTAAGGTAGCTCTCGGATCCGTTTATTGCGATTGGTTGTTTGT	
916h4-dnF	TATTCCATCTAAGCCATAGTACCCTCGAGATTCTCGATTAGCTGGTTTGAGAT	
916h4-dnR	TATTTCAGGTTGCGGGTCAGA	
916h4hph-F	refer to 4742h4hph-F	
916h4hph-R	refer to 4742h4hph-R	
916h4-F2	TCTGACCGCCAAACTCGACT	Verification of *hfb4* deletion in *T. harzianum* CBS 226.95
916h4-R2	refer to 4742h4-R2 (hph158 R)	
916h4-F3	refer to 4743h4 F3	
916h4-R3	refer to 4743h4 R3	
916h4-F4	refer to 4742h4-F4 (hph430 F)	
916h4-R4	AGGTCGAAAAAGACTGGGAAGT	

916h10-upF	GTAGGAATGGGATAAAGACAAGGAC	Deletion of *hfb10* in *T. harzianum* CBS 226.95
916h10-upR	CATATTGATGTAAGGTAGCTCTCGGATCCTAAGGAGTAGAGGTTTGGAGATGC	
916h10-dnF	TATTCCATCTAAGCCATAGTACCCTCGAGATGACTACCGAGGTCCTTTGATGAT	
916h10-dnR	TCGAGAAAAGCCATGGAGAGG	
916h10hph-F	refer to 4742h4hph-F	
916h10hph-R	refer to 4742h4hph-R	
916h10-F2	ATCGCCCCGCTATGATGCT	Verification of *hfb10* deletion in *T. harzianum* CBS 226.95
916h10-R2	refer to 4742h4-R2 (hph158 R)	
916h10-F3	TGCCGCACCAAGCAACTACT	
916h10-R3	AATAGCCGTCTGGCAAAGGA	
916h10-F4	refer to 4742h4-F4 (hph430 F)	
916h10-R4	ACAAGCCGGTCCTGCAGTT	
916h10neo-upF	TCTCAAGATTGGAGTAGGAATGG	Deletion of *hfb410* in $_{Th}\Delta$*hfb4*-3 mutant of *T. harzianum* CBS 226.95
916h10neo-upR	CTCGTATGACCTGAAGGAAATCAAAGCCGTAAGGAGTAGAGGTTTGGAGATGC	
916h10neo-dnF	CGCGACTATATATTTGTCTCTAATTGTACAAATGACTACCGAGGTCCTTTGAT	
916h10neo-dnR	GCTCATCACGTACTCGACTCTTG	
916h10neo-F	refer to 4742h10neo-F	
916h10neo-R	refer to 4742h10neo-R	
916h10neo-F2	refer to 916h10-F4	Verification of *hfb10* deletion in $_{Th}\Delta$*hfb4*-3 mutant
916h10neo-R2	refer to 4742h10neo-R2 (neo500 R)	
916h10neo-F3	refer to 916h10-F3	
916h10neo-R3	refer to 916h10-R3	
916h10neo-F4	refer to 4742h10neo-F4 (neo372 F)	
916h10neo-R4	refer to 916h10-R4	
hfb4seq-F	AGAACTGAGCCGACCGTCC	Cloning of *hfb4* from *Trichoderma* spp. for sequencing
hfb4seq-R	CCAGACAATCCAACTCCATCG	
hfb10seq-F	CACAACTCAGCATCCAACAGC	Cloning of *hfb10* from *Trichoderma* spp. for sequencing
hfb10seq-R	CATATGTTCAAGTCAGTGTCCAGTG	
4742qtef1-F	TACAAGATCGGTGGTATTGGAACA	Transcriptional quantification of *tef1* in *T. guizhouense* NJAU 4742
4742qtef1-R	AGCTGCTCGTGGTGCATCTC	
4742qhfb2-F	GGTCTCTTCTCCAACCCGCT	Transcriptional quantification of *hfb2* in *T. guizhouense* NJAU 4742
4742qhfb2-R	CTTGCCAATGGAAGCACAGTT	
4742qhfb3-F	CTTGGTGTCGCTGATGTCG	Transcriptional quantification of *hfb3* in *T. guizhouense* NJAU 4742
4742qhfb3-R	ATGGCATCGGTGCAGAGAA	
4742qhfb4-F	ACTCAGGAGCACCAGTTCGATA	Transcriptional quantification of *hfb4* in *T. guizhouense* NJAU 4742
4742qhfb4-R	TTCATCGGGAGTCTCGCAGT	
4742qhfb5-F	GACGATAGCGGCGATAGTGG	Transcriptional quantification of *hfb5* in *T. guizhouense* NJAU 4742
4742qhfb5-R	TCCTGTCTTTAGGCTCCTTGCT	
4742qhfb6-F	CATTGCCGACTTGGATTGTG	Transcriptional quantification of *hfb6* in *T. guizhouense* NJAU 4742
4742qhfb6-R	AAGAACTGGGATAGCACAGCAAC	
4742qhfb9a-F	TGGTGGCAAAGATGATGACG	Transcriptional quantification of *hfb9a* in *T. guizhouense* NJAU 4742
4742qhfb9a-R	ACCCTTCCCATTGCTACTGC	
4742qhfb9b-F	GCACGAACACTCAGGTAAACG	Transcriptional quantification of *hfb9b* in *T. guizhouense* NJAU 4742
4742qhfb9b-R	TGAAGTCGAGGTCGCCAAT	
4742qhfb10-F	CAATGCTGCTCTACCCTCGTT	Transcriptional quantification of *hfb10* in *T. guizhouense* NJAU 4742
4742qhfb10-R	CTTGGCAAAGGAGGTCTTGG	
4742qhfb15-F	AAAGGTAATGGCGATGGTAAAG	Transcriptional quantification of OPB45278 in *T. guizhouense* NJAU 4742
4742qhfb15-R	GCTGTTTCCGAGGATACCAATA	
916qtef1-F	Refer to 4742qtef1-F	

916qtef1-R	Refer to 4742qtef1-R	Transcriptional quantification of *tef1* in *T. harzianum* CBS 226.95
916qhfb1-F<br>916qhfb1-R	TCCTCTTGAGGATGCCCTCA<br>CGTTTGGAATATGGCAGTCG	Transcriptional quantification of PTB60449 in *T. harzianum* CBS 226.95
916qhfb2-F<br>916qhfb2-R	CTCTCTTCGTCGCCAGCG<br>GGAAGATGCCGACATTGGGT	Transcriptional quantification of *hfb2* in *T. harzianum* CBS 226.95
916qhfb3-F<br>916qhfb3-R	TCTTCACCGCAGCTTTCG<br>GGCGTGACGGGAGGAGAG	Transcriptional quantification of *hfb3* in *T. harzianum* CBS 226.95
916qhfb4-F<br>916qhfb4-R	ATGAAGTTCTCTGCCATCGCTC<br>TTCCTTGGGAGTCTCGCAGTT	Transcriptional quantification of *hfb4* in *T. harzianum* CBS 226.95
916qhfb5-F<br>916qhfb5-R	CGTCAAACCTCACCTTGTCCT<br>CAGCTACTGGGATAGCACAGCA	Transcriptional quantification of *hfb5* in *T. harzianum* CBS 226.95
916qhfb6-F<br>916qhfb6-R	TACGGCGGCTACTCTCCTGTT<br>ATCGCTACCGTCGTCGCTAT	Transcriptional quantification of *hfb6* in *T. harzianum* CBS 226.95
916qhfb9a-F<br>916qhfb9a-R	ACGCCTCTGTTATCCTTTACGC<br>TGATTGTAGTTGCGGGTTGG	Transcriptional quantification of *hfb9a* in *T. harzianum* CBS 226.95
916qhfb9b-F<br>916qhfb9b-R	TGCCTCTATCATCTTTTATGCTCTC<br>GCTGCTACCTTTACCCATCTTG	Transcriptional quantification of *hfb9b* in *T. harzianum* CBS 226.95
916qhfb10-F<br>916qhfb10-R	GGTAACGGCAACACCAACGA<br>TTAGCAGCACAGGCATTTTTGA	Transcriptional quantification of *hfb10* in *T. harzianum* CBS 226.95
916qhfb15-F<br>916qhfb15-R	TCACTTTGGGTGGTGCTTGC<br>CGGTGATTGTGACGACGACTG	Transcriptional quantification of PTB49111 in *T. harzianum* CBS 226.95
916qhfb17-F<br>916qhfb17-R	TCCTAAGCATCCTCGCTGTCA<br>TCTGGAATTGTTCAACCCCTG	Transcriptional quantification of PTB56946 in *T. harzianum* CBS 226.95

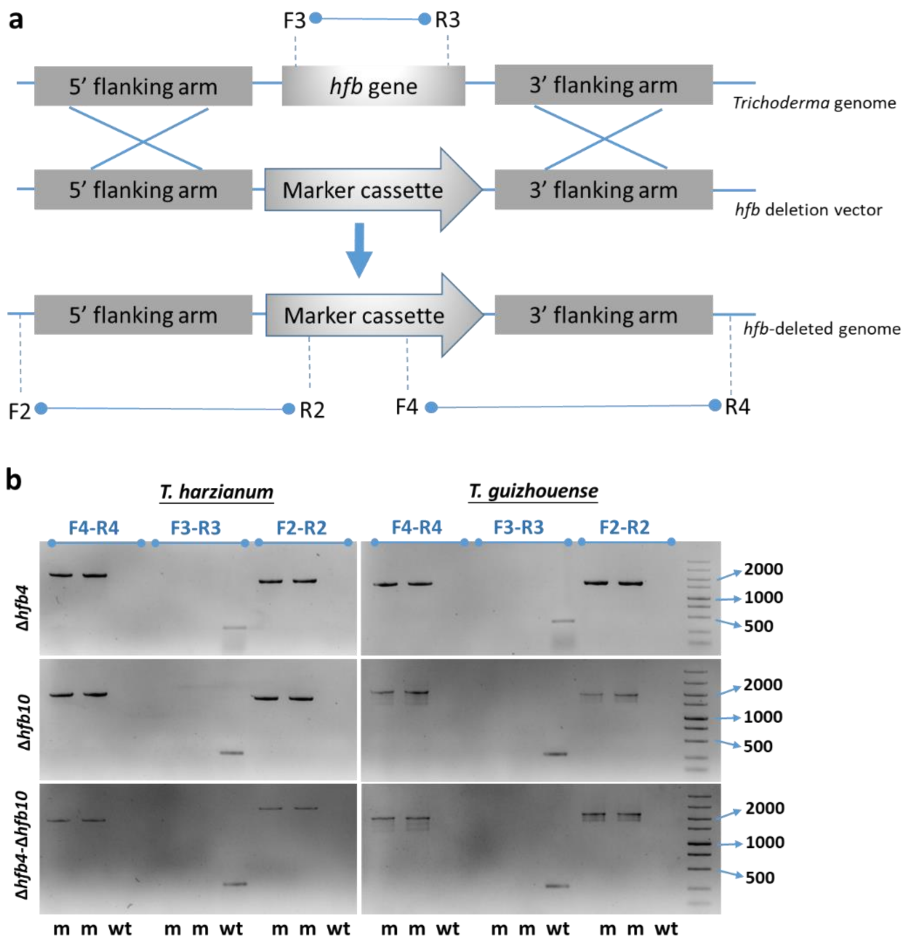

Fig. S1. Mutant construction and verification.

Schematic diagram of gene deletion via homologous recombination (**a**) and mutant verification by PCR (**b**). wt represents the corresponding wild type strains; m represents mutants. DL5000 DNA marker (Vazyme, China) was used in gel electrophoresis. Genes of *hfb* in *T. harzianum* CBS 226.95 (GenBank: MBGI00000000.1, (Kubicek et al 2019)) and *T. guizhouense* NJAU 4742 (GenBank: LVVK00000000.1, (Kubicek et al 2019)) were obtained by genome mining. Gene IDs were shown as in **Fig. 1.** Vectors for single gene deletion was constructed as shown in **a** (see primers in **Table S2**). For the double deletion strains, *hfb4* gene was replaced by *hph* cassette (Uzbas et al 2012) and *hfb10* gene was replaced by *neo* cassette (Seiboth et al 2012) with two times of transformation. Putative transformants were first screened for the presence of the marker gene right at the downstream of the 5′ flanking arm with the primer pair F2 and R2. The positive ones were then screened for double exchange (homologous recombination) with the primer pair F4 and R4. Positive mutants were purified by the method of single spore isolation and confirmed for the absence of the target gene with the primer pair F3 and R3. All vectors and PCR products were confirmed by sequencing; similarly hereinafter. After purification and verification by PCR (shown in **b** with two randomly selected mutants), four $_{\mathrm{Th}}\Delta$*hfb4*, four $_{\mathrm{Th}}\Delta$*hfb10* and two $_{\mathrm{Th}}\Delta$*hfb4*$_{\mathrm{Th}}\Delta$*hfb10* were obtained for *T. harzianum* CBS 226.95 and four $_{\mathrm{Tg}}\Delta$*hfb4*, four $_{\mathrm{Tg}}\Delta$*hfb10* and two $_{\mathrm{Tg}}\Delta$*hfb4*$_{\mathrm{Tg}}\Delta$*hfb10* mutants were obtained for *T. guizhouense* NJAU 4742 (see strains used in this study in **Table S1**).

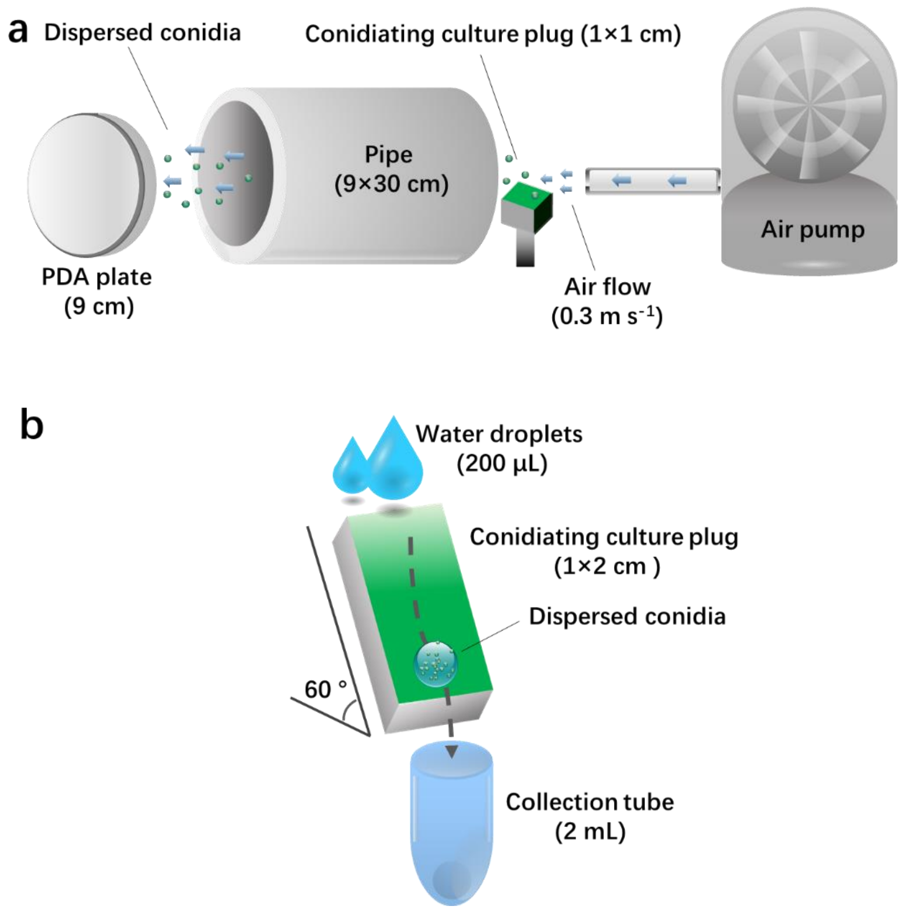

Fig. S2. Schematic diagram of experimental design for air (a) and water (b) dispersal tests.

In **a**, fungal culture covered by conidia were cropped into 1 × 1 cm plugs and blown under a constant air flow (0.3 m s^{-1}) through a 30 cm-long pipe. Spores carried away from the parental body were then trapped by a 9 cm PDA plate installed on the opposite end of the pipe, and colony forming unites (CFU) on the plate were counted after incubation to estimate the air dispersal ability for each strain. In **b**, the amount of spores that could be transported by water droplets was measured by releasing 200 µL of water along a 1 × 2 cm conidiating culture plug inclined at an angle of 60 °. The water dispersal ability was then expressed as the amount of the collected spores per µL of water that measured by a Biolog turbidity meter and transferred into number by a hemocytometer. The conidia abundance was calibrated by measuring the O.D. 590 nm of the culture plugs from the same plate for each of the above test.

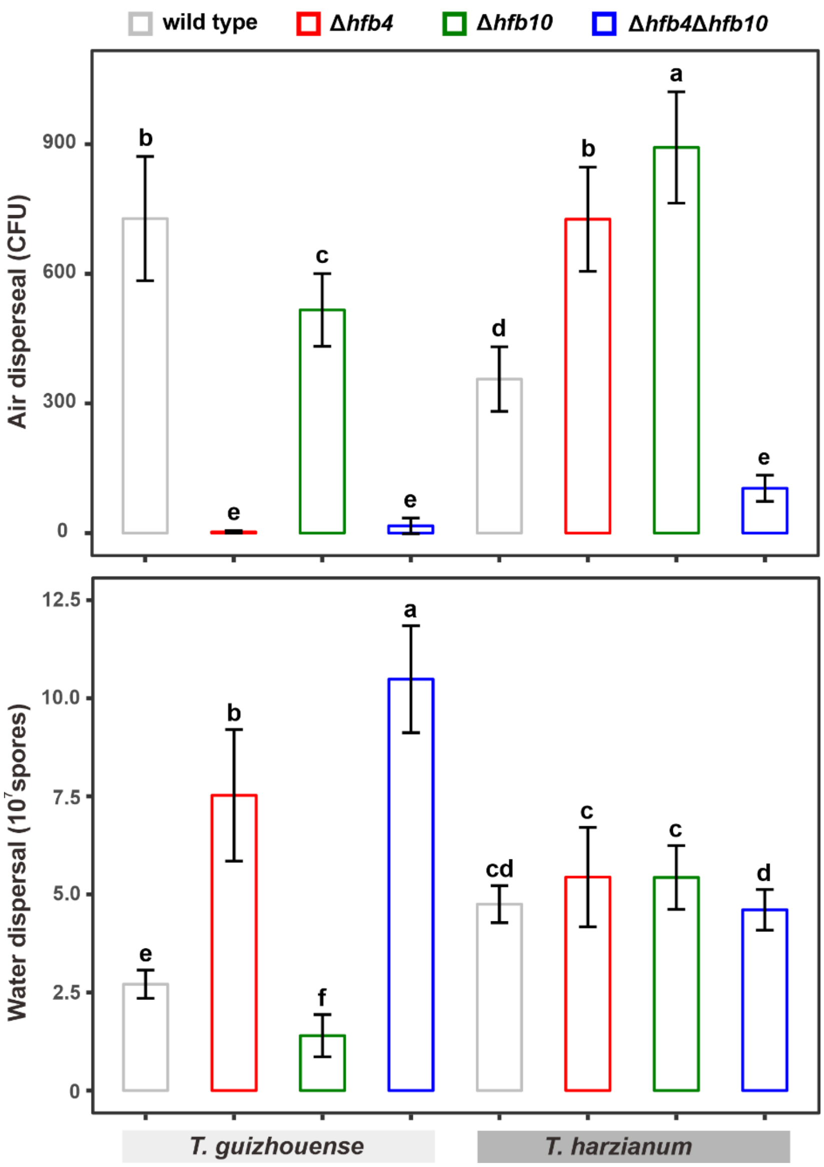

Fig. S3. Comparison of the ability of spores able to be dispersed by air or water between strains

The air dispersal ability (anemophily) of spores was expressed as the number of CFU formed by spores trapped from a 0.3 m s^{-1} air flow, and the water dispersal ability (pluviophilous) of spores were measured by the amount of spores could be transported by 200 µL of water droplets. Bars with different letters represent a statistically significant difference from each other at the level of $P < 0.05$.

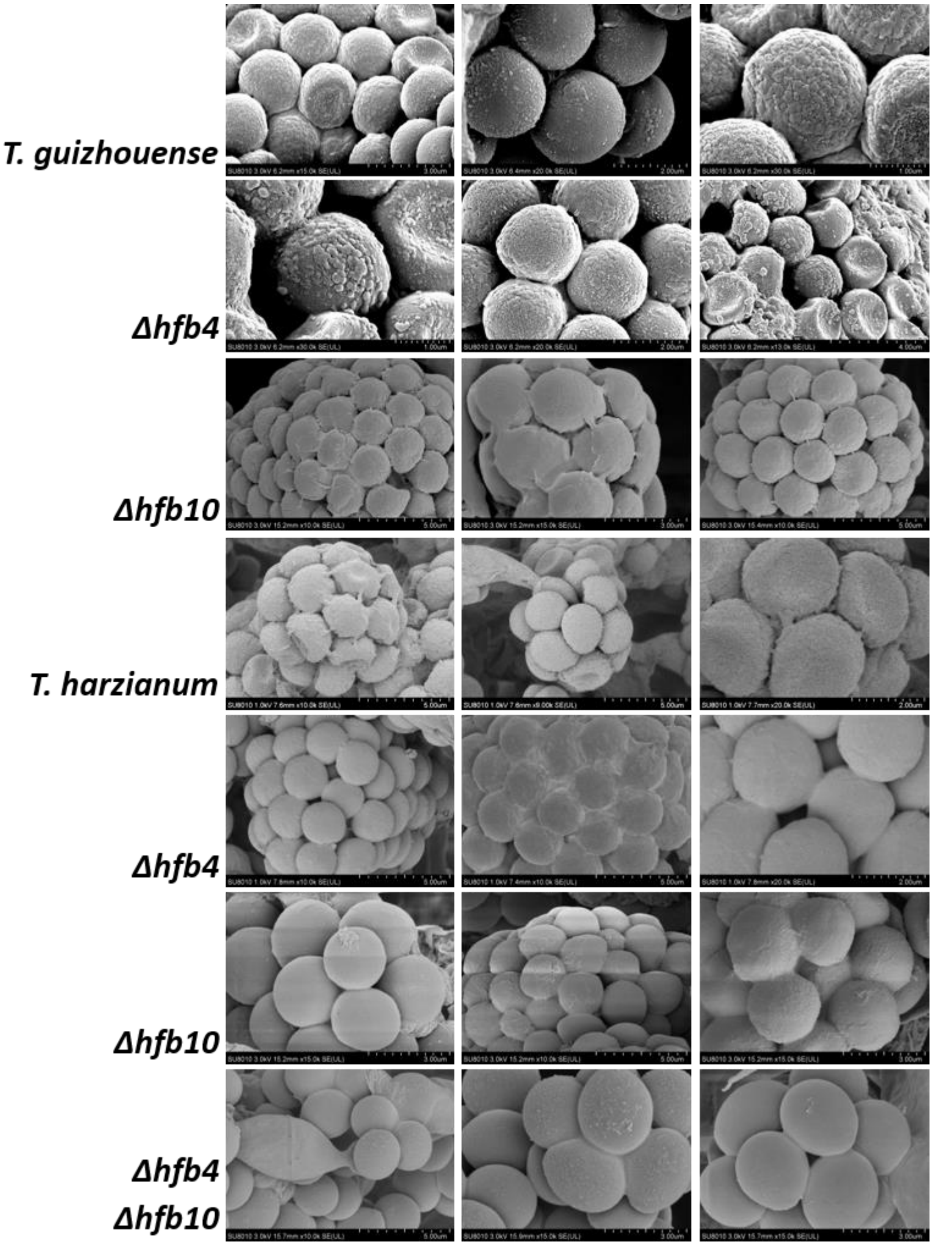

Fig. S4. Micromorphology of spores.

Fungal colonies were investigated by cryo-scanning electron microscopy (SEM, Quorum PP3010T integrated onto a Hitachi SU8010 FE-SEM, Japan). The spore size of *T. harzianum* and *T. guizhouense* wild type and *hfb* deletion strains was 2.4 (± 0.2) µm as estimated from 197 measurements.

a

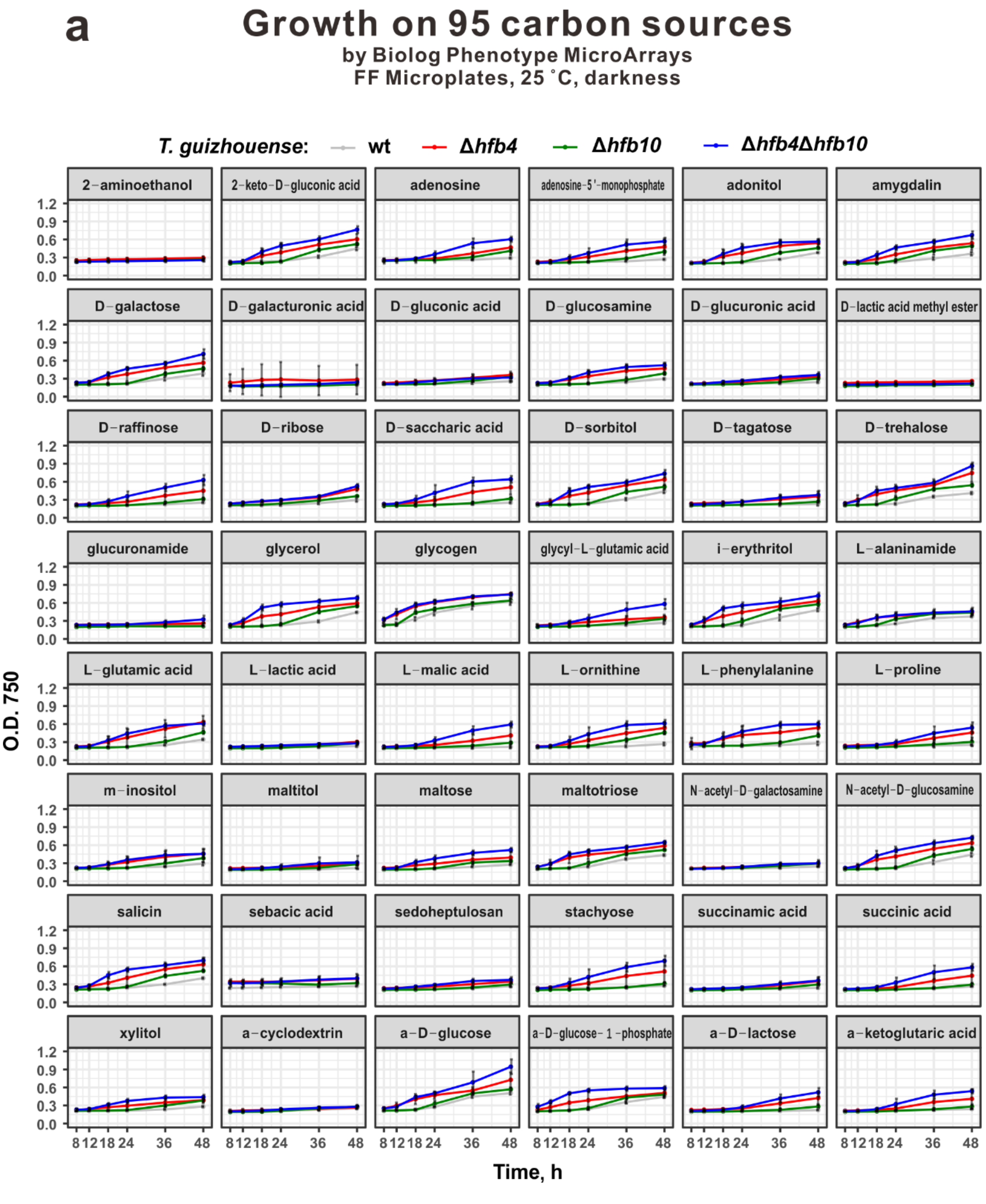
Growth on 95 carbon sources
by Biolog Phenotype MicroArrays
FF Microplates, 25 ˚C, darkness
T. guizhouense:
wt
Δhfb4
Δhfb10
Δhfb4Δhfb10
2−aminoethanol
2−keto−D−gluconic acid
adenosine
adenosine−5'−monophosphate
adonitol
amygdalin
D−galactose
D−galacturonic acid
D−gluconic acid
D−glucosamine
D−glucuronic acid
D−lactic acid methyl ester
D−raffinose
D−ribose
D−saccharic acid
D−sorbitol
D−tagatose
D−trehalose
glucuronamide
glycerol
glycogen
glycyl−L−glutamic acid
i−erythritol
L−alaninamide
L−glutamic acid
L−lactic acid
L−malic acid
L−ornithine
L−phenylalanine
L−proline
m−inositol
maltitol
maltose
maltotriose
N−acetyl−D−galactosamine
N−acetyl−D−glucosamine
salicin
sebacic acid
sedoheptulosan
stachyose
succinamic acid
succinic acid
xylitol
a−cyclodextrin
a−D−glucose
a−D−glucose−1−phosphate
a−D−lactose
a−ketoglutaric acid
O.D. 750
Time, h

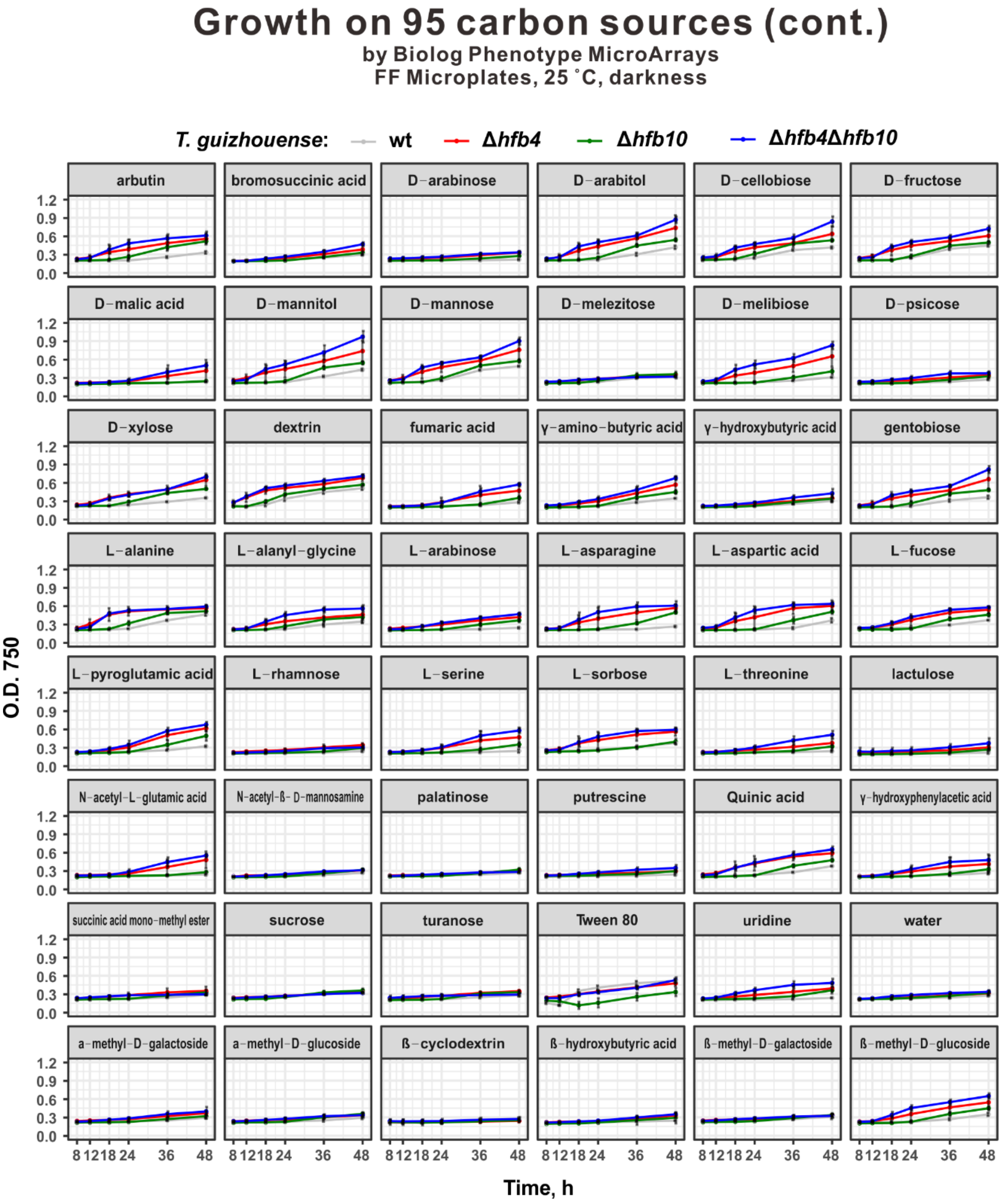
Growth on 95 carbon sources (cont.)
by Biolog Phenotype MicroArrays
FF Microplates, 25 ˚C, darkness
T. guizhouense: wt Δhfb4 Δhfb10 Δhfb4Δhfb10
arbutin
bromosuccinic acid
D−arabinose
D−arabitol
D−cellobiose
D−fructose
D−malic acid
D−mannitol
D−mannose
D−melezitose
D−melibiose
D−psicose
D−xylose
dextrin
fumaric acid
γ−amino−butyric acid
γ−hydroxybutyric acid
gentobiose
L−alanine
L−alanyl−glycine
L−arabinose
L−asparagine
L−aspartic acid
L−fucose
L−pyroglutamic acid
L−rhamnose
L−serine
L−sorbose
L−threonine
lactulose
N−acetyl−L−glutamic acid
N−acetyl−ß− D−mannosamine
palatinose
putrescine
Quinic acid
γ−hydroxyphenylacetic acid
succinic acid mono−methyl ester
sucrose
turanose
Tween 80
uridine
water
a−methyl−D−galactoside
a−methyl−D−glucoside
ß−cyclodextrin
ß−hydroxybutyric acid
ß−methyl−D−galactoside
ß−methyl−D−glucoside
O.D. 750
0.0
0.3
0.6
0.9
1.2
8 12 18 24 36 48
Time, h

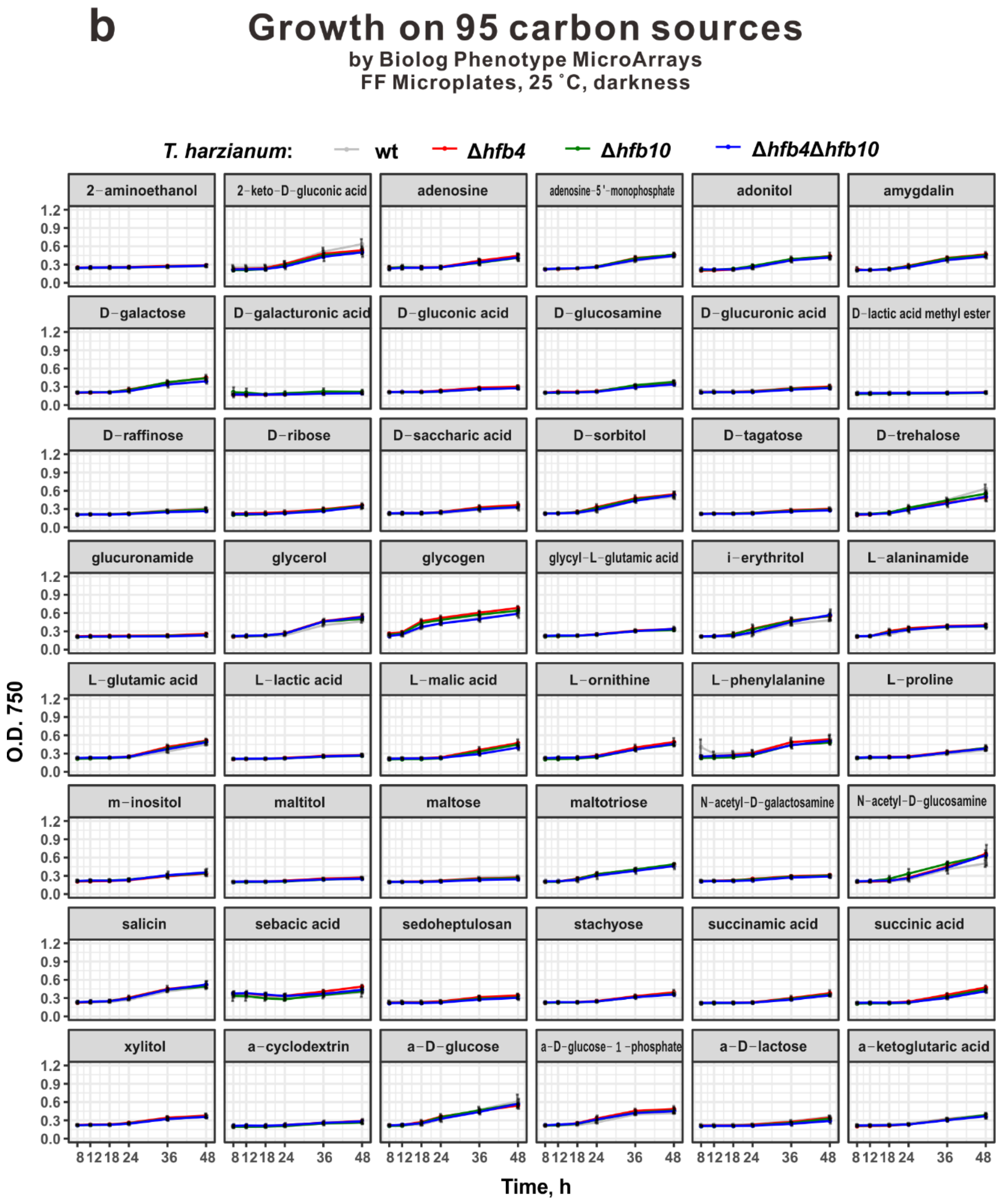
b
Growth on 95 carbon sources
by Biolog Phenotype MicroArrays
FF Microplates, 25 ˚C, darkness
T. harzianum:
wt
Δhfb4
Δhfb10
Δhfb4Δhfb10
2−aminoethanol
2−keto−D−gluconic acid
adenosine
adenosine−5'−monophosphate
adonitol
amygdalin
D−galactose
D−galacturonic acid
D−gluconic acid
D−glucosamine
D−glucuronic acid
D−lactic acid methyl ester
D−raffinose
D−ribose
D−saccharic acid
D−sorbitol
D−tagatose
D−trehalose
glucuronamide
glycerol
glycogen
glycyl−L−glutamic acid
i−erythritol
L−alaninamide
L−glutamic acid
L−lactic acid
L−malic acid
L−ornithine
L−phenylalanine
L−proline
m−inositol
maltitol
maltose
maltotriose
N−acetyl−D−galactosamine
N−acetyl−D−glucosamine
salicin
sebacic acid
sedoheptulosan
stachyose
succinamic acid
succinic acid
xylitol
a−cyclodextrin
a−D−glucose
a−D−glucose−1−phosphate
a−D−lactose
a−ketoglutaric acid
O.D. 750
0.0
0.3
0.6
0.9
1.2
8 12 18 24 36 48
Time, h

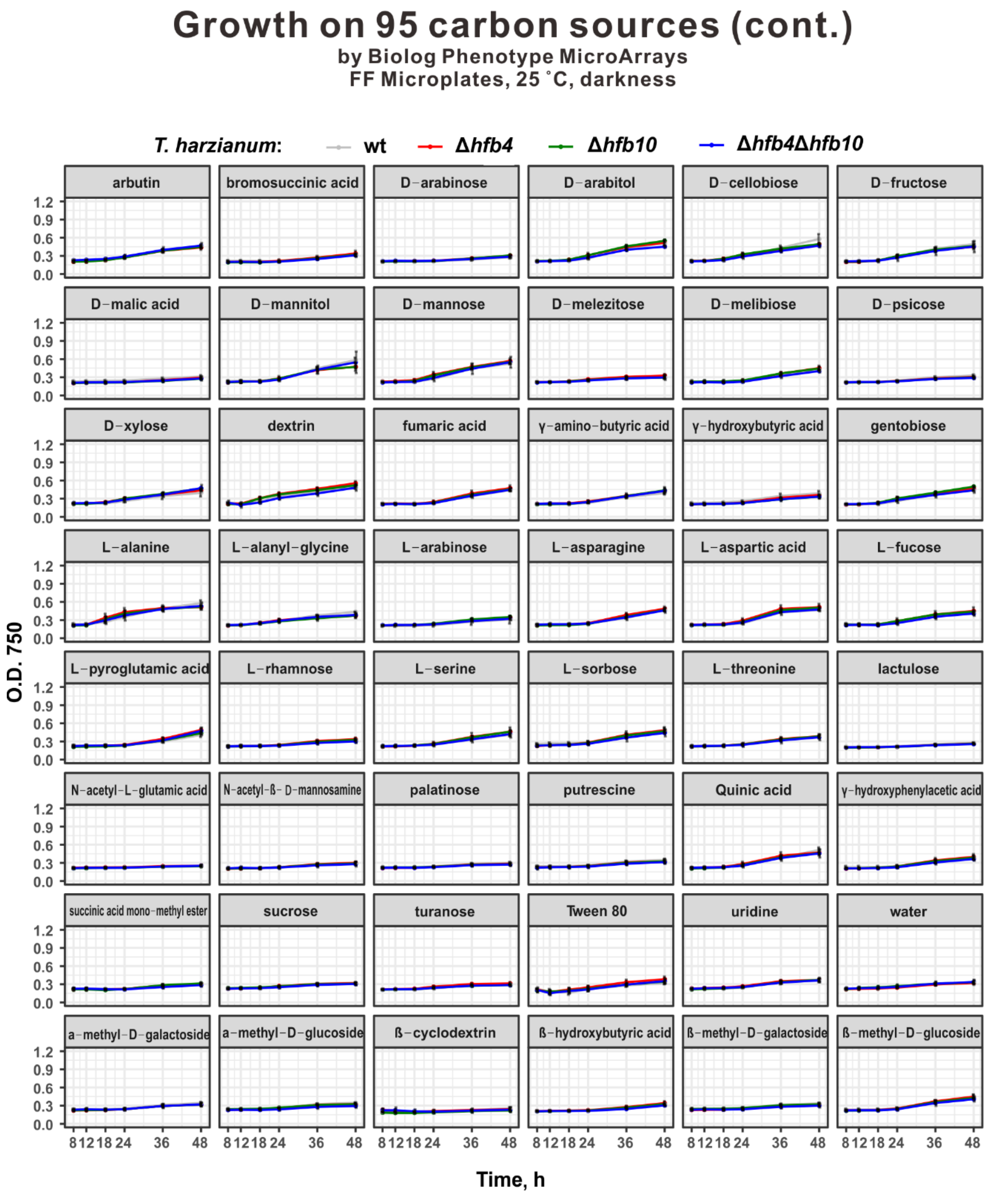

Fig. S5. The role of HFBs in regulating growth of *T. guizhouense* (a) and *T. harzianum* (b).

The line plots showing the vegetative growth (shown by the values of O.D.750 nm) of each genotype grown on 95 carbon sources in Biolog FF Microplates. Two mutants were used for each haplotype. Error bars represent the standard deviations calculated from at least three replicates.

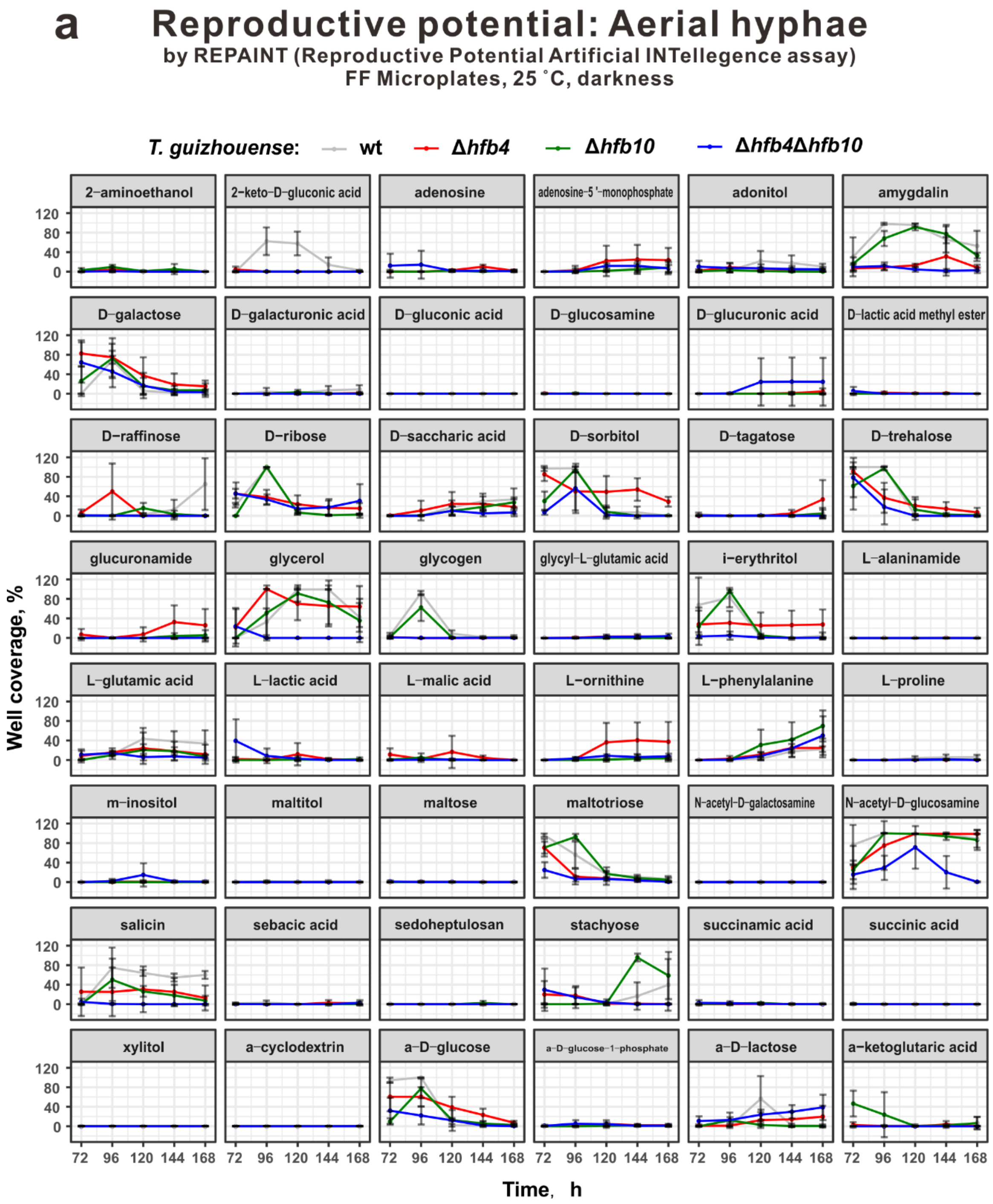
a
Reproductive potential: Aerial hyphae
by REPAINT (Reproductive Potential Artificial INTellegence assay)
FF Microplates, 25 ˚C, darkness
T. guizhouense: wt Δhfb4 Δhfb10 Δhfb4Δhfb10
2−aminoethanol
2−keto−D−gluconic acid
adenosine
adenosine−5 '−monophosphate
adonitol
amygdalin
D−galactose
D−galacturonic acid
D−gluconic acid
D−glucosamine
D−glucuronic acid
D−lactic acid methyl ester
D−raffinose
D−ribose
D−saccharic acid
D−sorbitol
D−tagatose
D−trehalose
glucuronamide
glycerol
glycogen
glycyl−L−glutamic acid
i−erythritol
L−alaninamide
L−glutamic acid
L−lactic acid
L−malic acid
L−ornithine
L−phenylalanine
L−proline
m−inositol
maltitol
maltose
maltotriose
N−acetyl−D−galactosamine
N−acetyl−D−glucosamine
salicin
sebacic acid
sedoheptulosan
stachyose
succinamic acid
succinic acid
xylitol
a−cyclodextrin
a−D−glucose
a−D−glucose−1−phosphate
a−D−lactose
a−ketoglutaric acid
Well coverage, %
120
80
40
0
72 96 120 144 168
Time, h

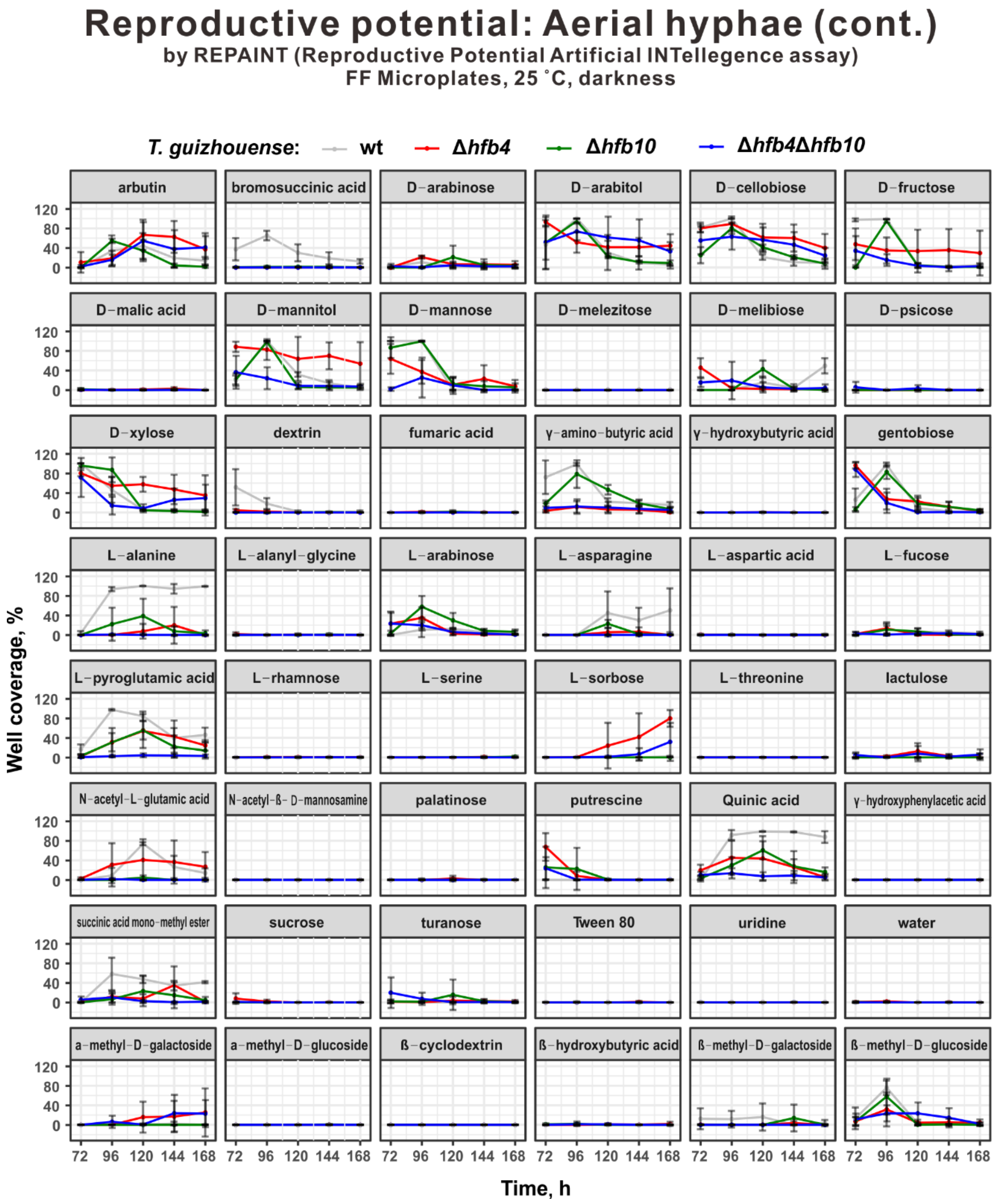
Reproductive potential: Aerial hyphae (cont.)
by REPAINT (Reproductive Potential Artificial INTellegence assay)
FF Microplates, 25 ˚C, darkness
T. guizhouense: wt Δhfb4 Δhfb10 Δhfb4Δhfb10
arbutin
bromosuccinic acid
D−arabinose
D−arabitol
D−cellobiose
D−fructose
D−malic acid
D−mannitol
D−mannose
D−melezitose
D−melibiose
D−psicose
D−xylose
dextrin
fumaric acid
γ−amino−butyric acid
γ−hydroxybutyric acid
gentobiose
L−alanine
L−alanyl−glycine
L−arabinose
L−asparagine
L−aspartic acid
L−fucose
L−pyroglutamic acid
L−rhamnose
L−serine
L−sorbose
L−threonine
lactulose
N−acetyl−L−glutamic acid
N−acetyl−ß− D−mannosamine
palatinose
putrescine
Quinic acid
γ−hydroxyphenylacetic acid
succinic acid mono−methyl ester
sucrose
turanose
Tween 80
uridine
water
a−methyl−D−galactoside
a−methyl−D−glucoside
ß−cyclodextrin
ß−hydroxybutyric acid
ß−methyl−D−galactoside
ß−methyl−D−glucoside
Well coverage, %
Time, h

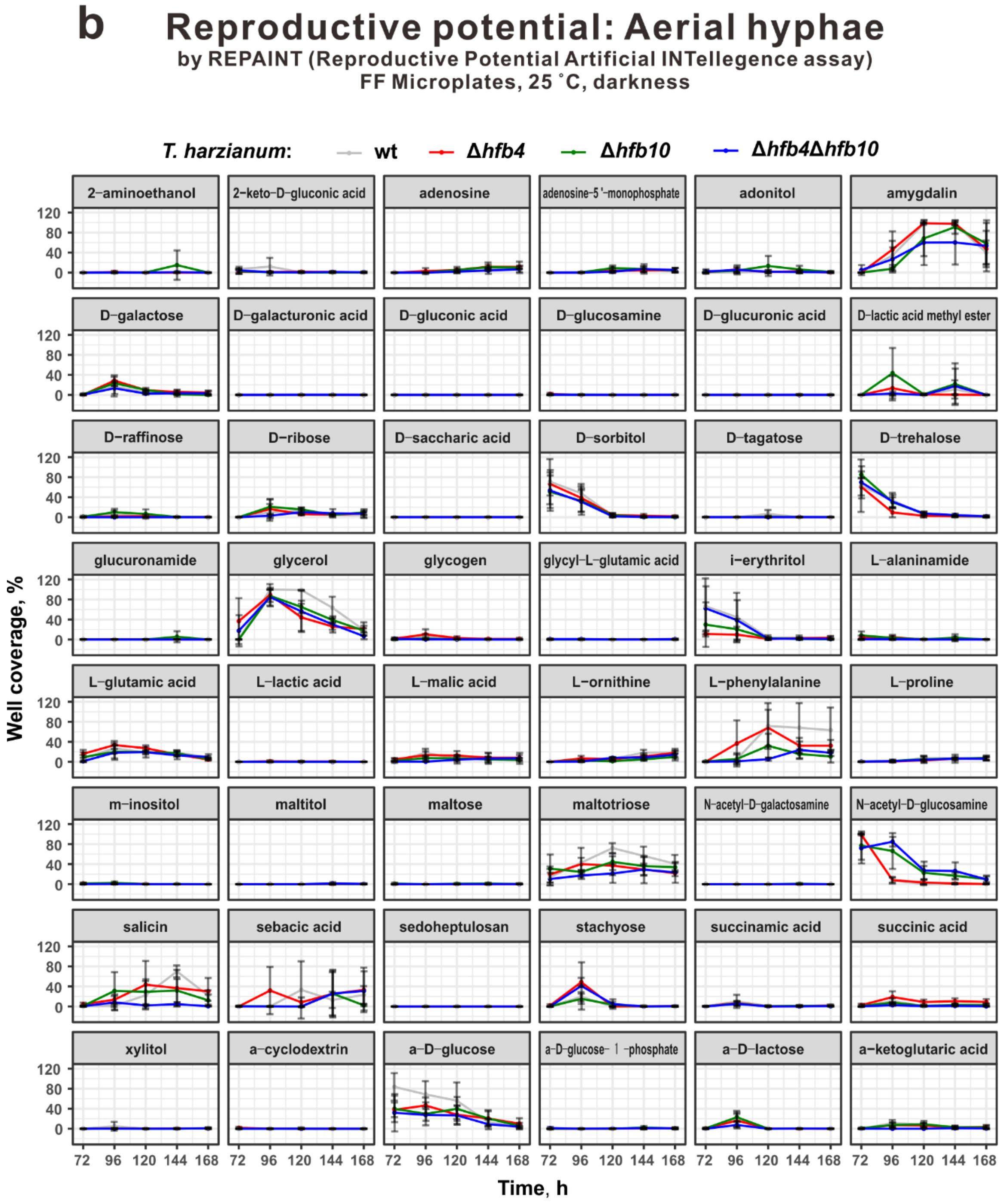
b
Reproductive potential: Aerial hyphae
by REPAINT (Reproductive Potential Artificial INTellegence assay)
FF Microplates, 25 ˚C, darkness
T. harzianum:
wt
Δhfb4
Δhfb10
Δhfb4Δhfb10
2–aminoethanol
2–keto–D–gluconic acid
adenosine
adenosine–5 '–monophosphate
adonitol
amygdalin
D–galactose
D–galacturonic acid
D–gluconic acid
D–glucosamine
D–glucuronic acid
D–lactic acid methyl ester
D–raffinose
D–ribose
D–saccharic acid
D–sorbitol
D–tagatose
D–trehalose
glucuronamide
glycerol
glycogen
glycyl–L–glutamic acid
i–erythritol
L–alaninamide
L–glutamic acid
L–lactic acid
L–malic acid
L–ornithine
L–phenylalanine
L–proline
m–inositol
maltitol
maltose
maltotriose
N–acetyl–D–galactosamine
N–acetyl–D–glucosamine
salicin
sebacic acid
sedoheptulosan
stachyose
succinamic acid
succinic acid
xylitol
a–cyclodextrin
a–D–glucose
a–D–glucose– 1 –phosphate
a–D–lactose
a–ketoglutaric acid
Well coverage, %
0
40
80
120
72
96
120
144
168
Time, h

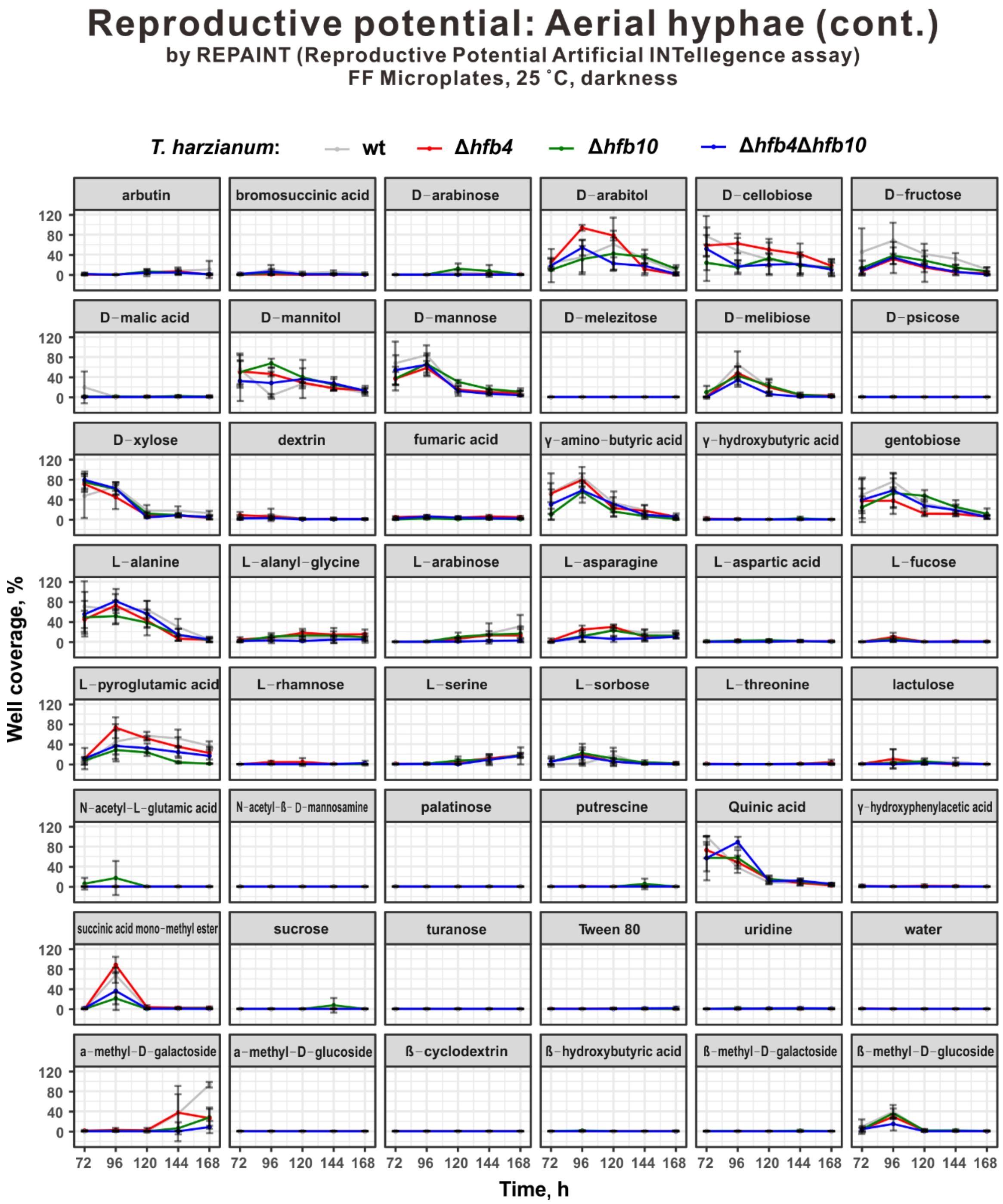

Fig. S6. The role of HFBs in regulating aerial hypha formation of *T. guizhouense* (a) and *T. harzianum* (b).

The line plots showing the development of aerial hypha (shown as % coverage per well) of each genotype grown on 95 carbon sources in Biolog FF Microplates. Two mutants were used for each haplotype. Error bars represent the standard deviations calculated from at least three replicates.

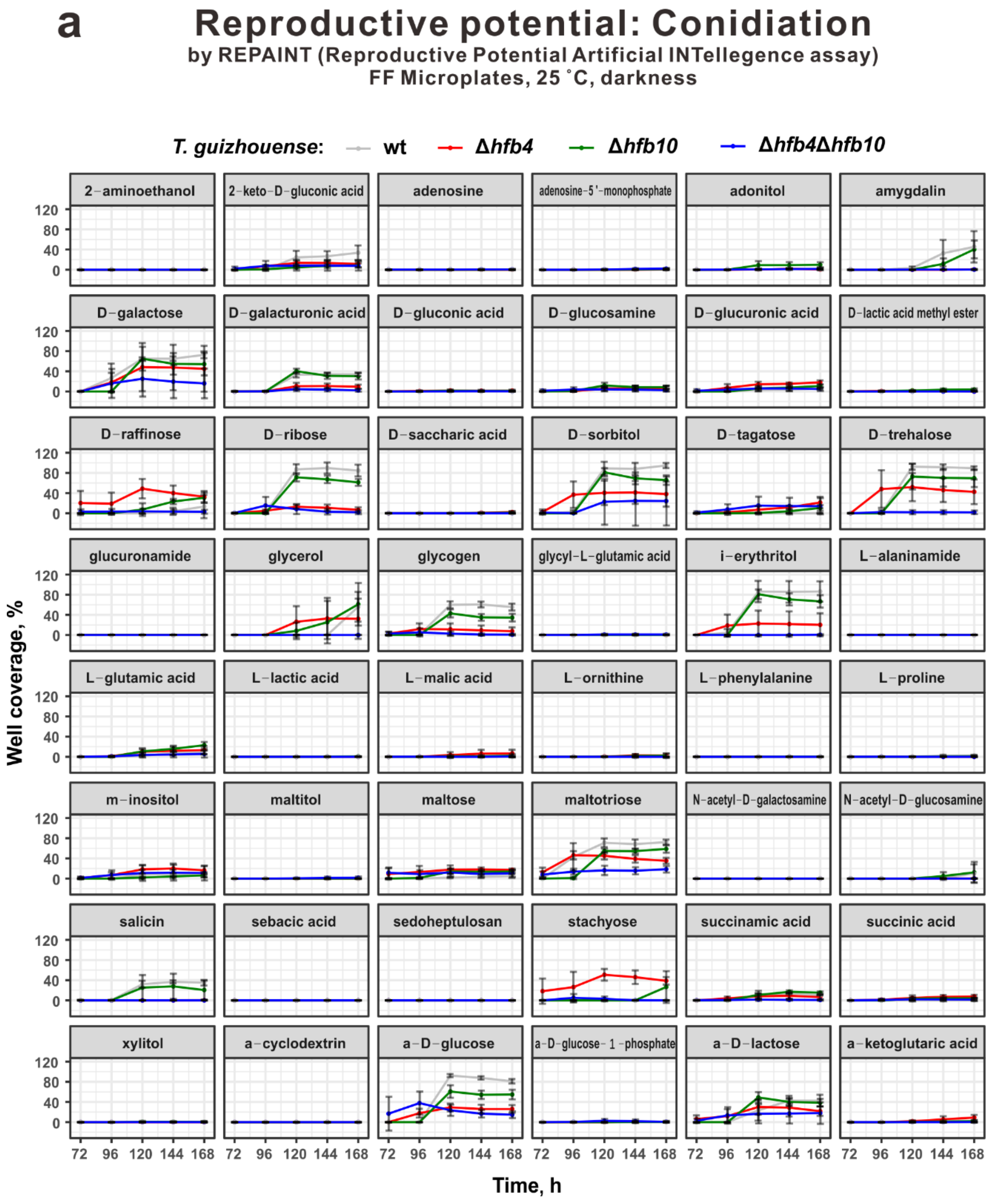
a
Reproductive potential: Conidiation
by REPAINT (Reproductive Potential Artificial INTellegence assay)
FF Microplates, 25 °C, darkness
T. guizhouense: wt Δhfb4 Δhfb10 Δhfb4Δhfb10
2−aminoethanol
2−keto−D−gluconic acid
adenosine
adenosine−5'−monophosphate
adonitol
amygdalin
D−galactose
D−galacturonic acid
D−gluconic acid
D−glucosamine
D−glucuronic acid
D−lactic acid methyl ester
D−raffinose
D−ribose
D−saccharic acid
D−sorbitol
D−tagatose
D−trehalose
glucuronamide
glycerol
glycogen
glycyl−L−glutamic acid
i−erythritol
L−alaninamide
L−glutamic acid
L−lactic acid
L−malic acid
L−ornithine
L−phenylalanine
L−proline
m−inositol
maltitol
maltose
maltotriose
N−acetyl−D−galactosamine
N−acetyl−D−glucosamine
salicin
sebacic acid
sedoheptulosan
stachyose
succinamic acid
succinic acid
xylitol
a−cyclodextrin
a−D−glucose
a−D−glucose−1−phosphate
a−D−lactose
a−ketoglutaric acid
Well coverage, %
0 40 80 120
72 96 120 144 168
Time, h

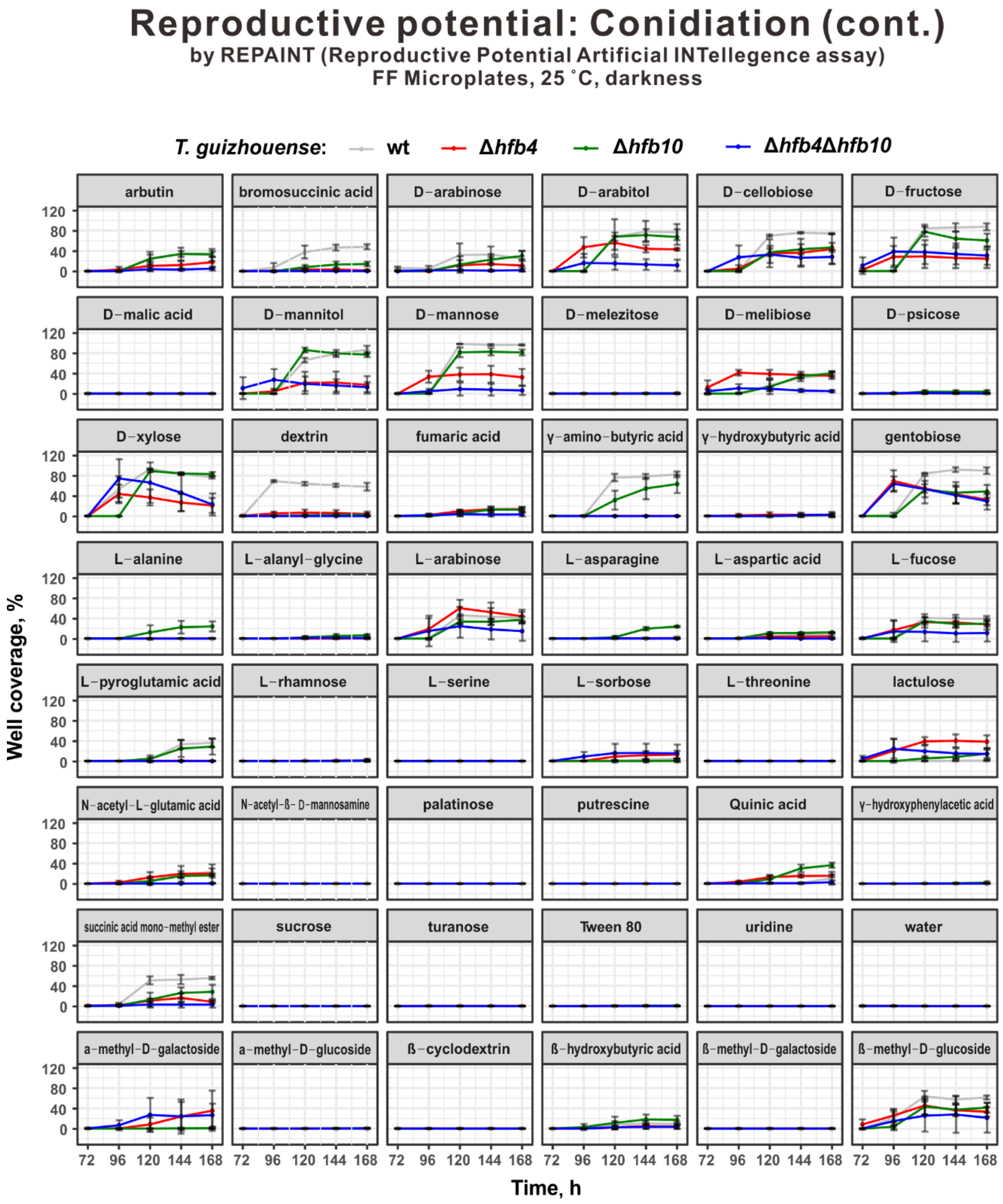
Reproductive potential: Conidiation (cont.)
by REPAINT (Reproductive Potential Artificial INTellegence assay)
FF Microplates, 25 ˚C, darkness
T. guizhouense: wt Δhfb4 Δhfb10 Δhfb4Δhfb10
arbutin
bromosuccinic acid
D−arabinose
D−arabitol
D−cellobiose
D−fructose
D−malic acid
D−mannitol
D−mannose
D−melezitose
D−melibiose
D−psicose
D−xylose
dextrin
fumaric acid
γ−amino−butyric acid
γ−hydroxybutyric acid
gentobiose
L−alanine
L−alanyl−glycine
L−arabinose
L−asparagine
L−aspartic acid
L−fucose
L−pyroglutamic acid
L−rhamnose
L−serine
L−sorbose
L−threonine
lactulose
N−acetyl−L−glutamic acid
N−acetyl−ß− D−mannosamine
palatinose
putrescine
Quinic acid
γ−hydroxyphenylacetic acid
succinic acid mono−methyl ester
sucrose
turanose
Tween 80
uridine
water
a−methyl−D−galactoside
a−methyl−D−glucoside
ß−cyclodextrin
ß−hydroxybutyric acid
ß−methyl−D−galactoside
ß−methyl−D−glucoside
Well coverage, %
0 40 80 120
72 96 120 144 168
Time, h

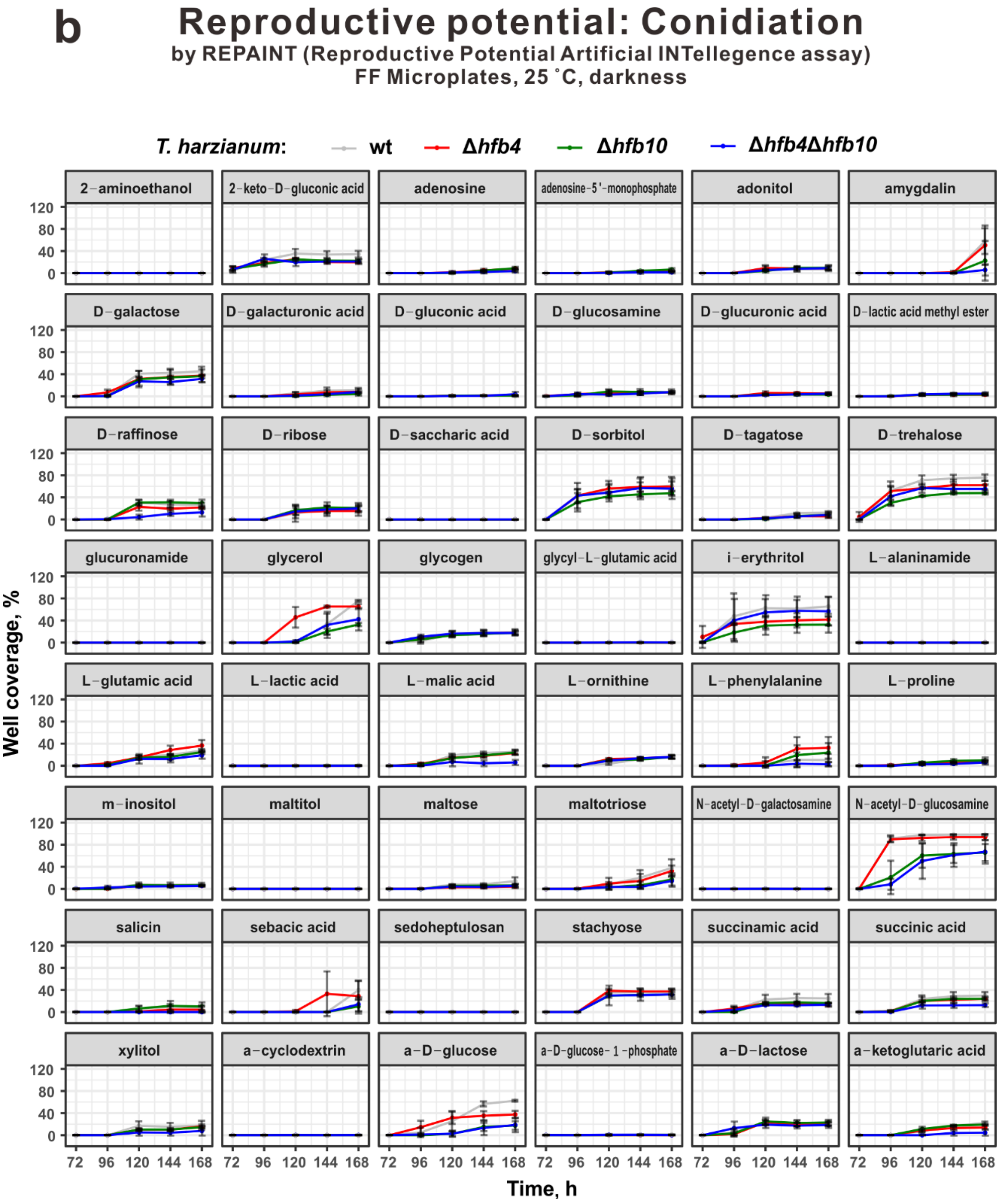
b
Reproductive potential: Conidiation
by REPAINT (Reproductive Potential Artificial INTellegence assay)
FF Microplates, 25 ˚C, darkness
T. harzianum:
wt
Δhfb4
Δhfb10
Δhfb4Δhfb10
2−aminoethanol
2−keto−D−gluconic acid
adenosine
adenosine−5'−monophosphate
adonitol
amygdalin
D−galactose
D−galacturonic acid
D−gluconic acid
D−glucosamine
D−glucuronic acid
D−lactic acid methyl ester
D−raffinose
D−ribose
D−saccharic acid
D−sorbitol
D−tagatose
D−trehalose
glucuronamide
glycerol
glycogen
glycyl−L−glutamic acid
i−erythritol
L−alaninamide
L−glutamic acid
L−lactic acid
L−malic acid
L−ornithine
L−phenylalanine
L−proline
m−inositol
maltitol
maltose
maltotriose
N−acetyl−D−galactosamine
N−acetyl−D−glucosamine
salicin
sebacic acid
sedoheptulosan
stachyose
succinamic acid
succinic acid
xylitol
a−cyclodextrin
a−D−glucose
a−D−glucose−1−phosphate
a−D−lactose
a−ketoglutaric acid
Well coverage, %
0
40
80
120
72
96
120
144
168
Time, h

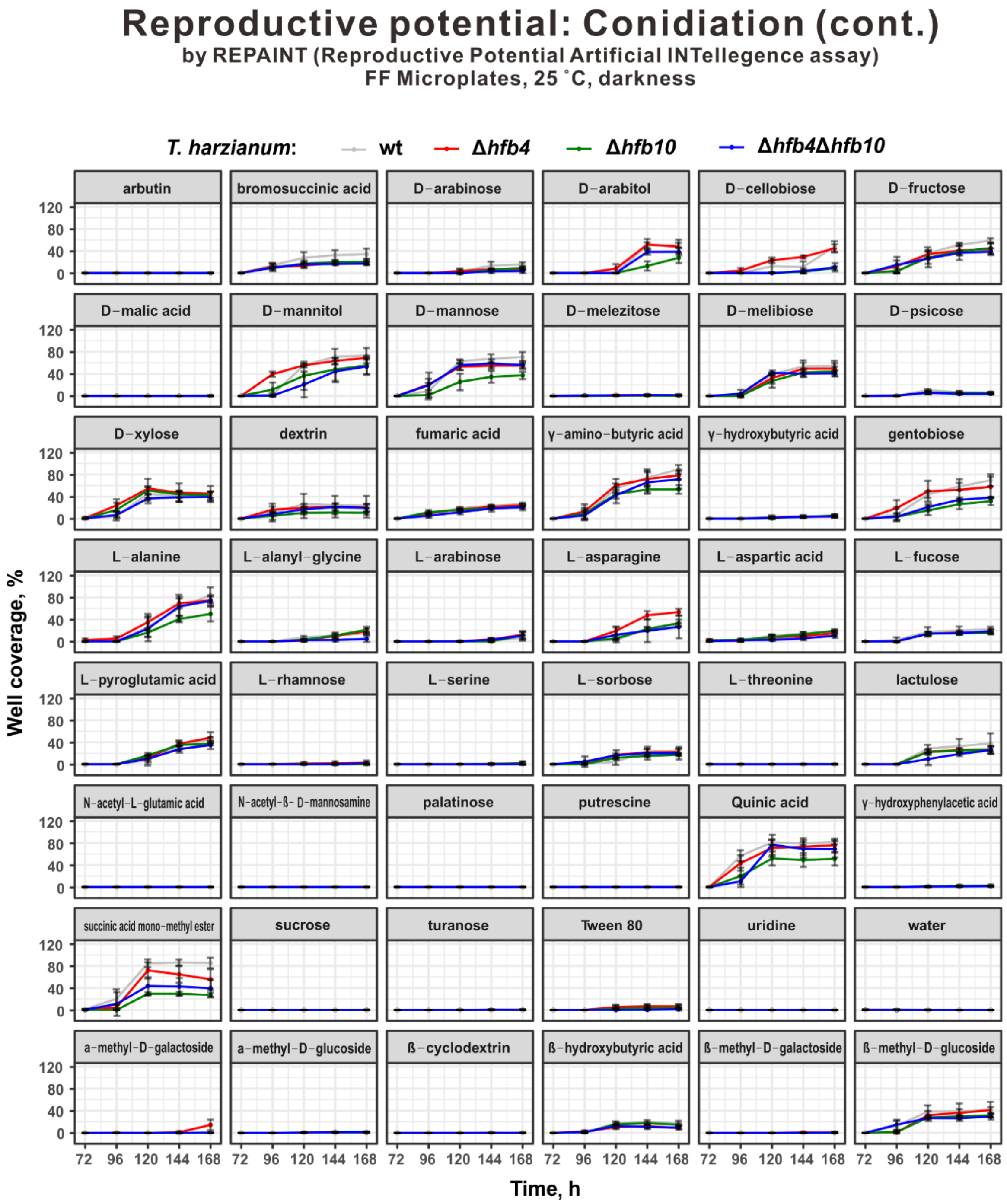

Fig. S7. The role of HFBs in regulating conidiation of *T. guizhouense* (a) and *T. harzianum* (b).

The line plots showing the conidia abundance (shown as % coverage per well) of each genotype grown on 95 carbon sources in Biolog FF Microplates. Two mutants were used for each haplotype. Error bars represent the standard deviations calculated from at least three replicates.

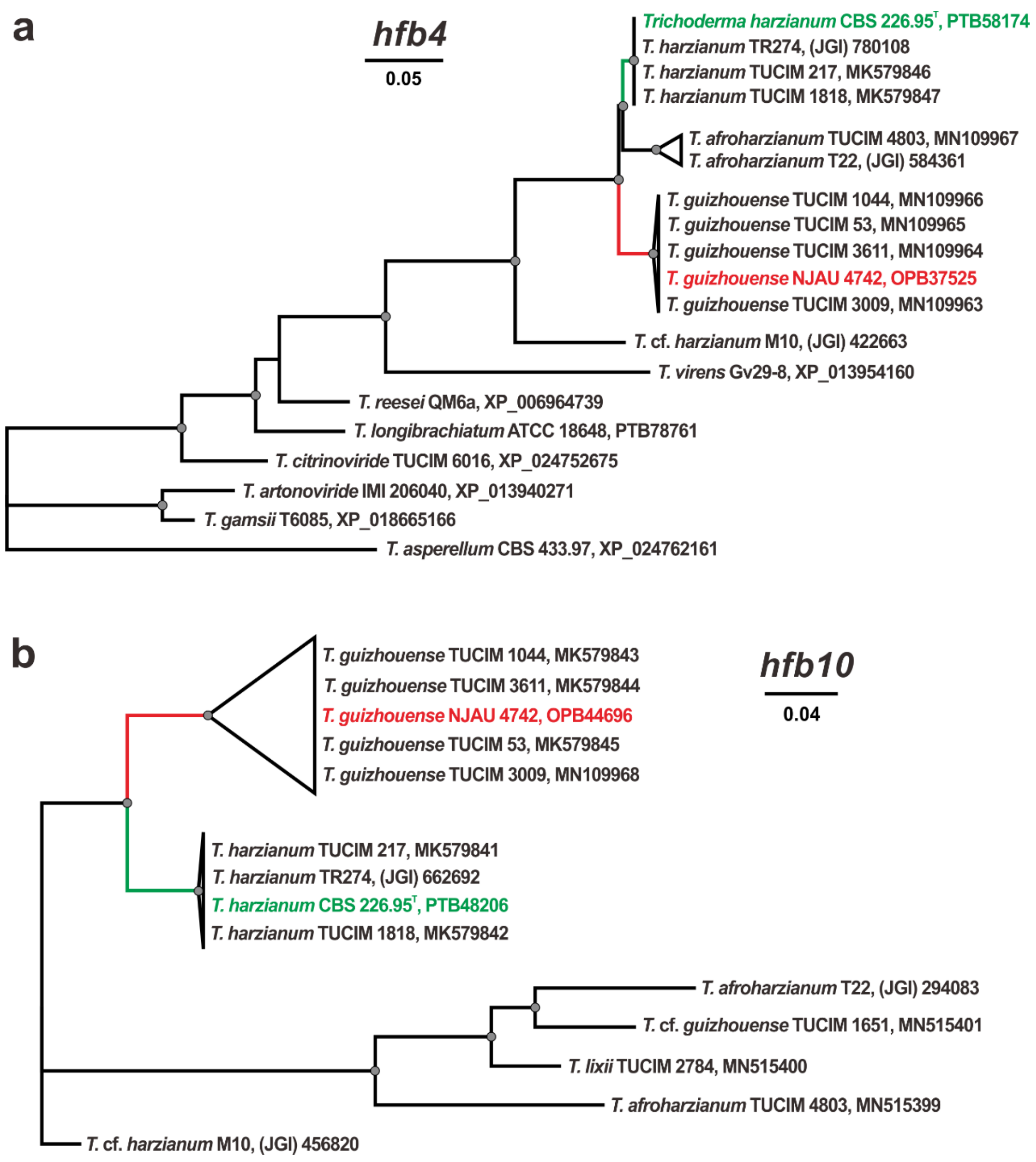

Fig. S8. Maximum likelihood phylogenetic trees of *hfb4* (a) and *hfb10* (b) genes from *T. guizhouense*, *T. harzianum* and their closely-related species.

Phylograms were constructed by IQ-TREE 1.6.12 (Nguyen et al 2015). Circles abode nodes indicate IQTree ultrafast bootstrap support values >60. The lineages of *T. guizhouense* (red) and *T. harzianum* (green) were respectively used as the foreground branches in the selection pressure analysis. The model strains used in the genetic work were also respectively marked as above. Monophyletic species clades were collapsed. Sequences were retrieved from the DOE Joint Genome Institute (JGI, shown with brackets) database or obtained by sequencing and deposited in the National Center for Biotechnology Information (NCBI) database. Gene accession numbers in public databases were given after the strain name. T, type strain.

a

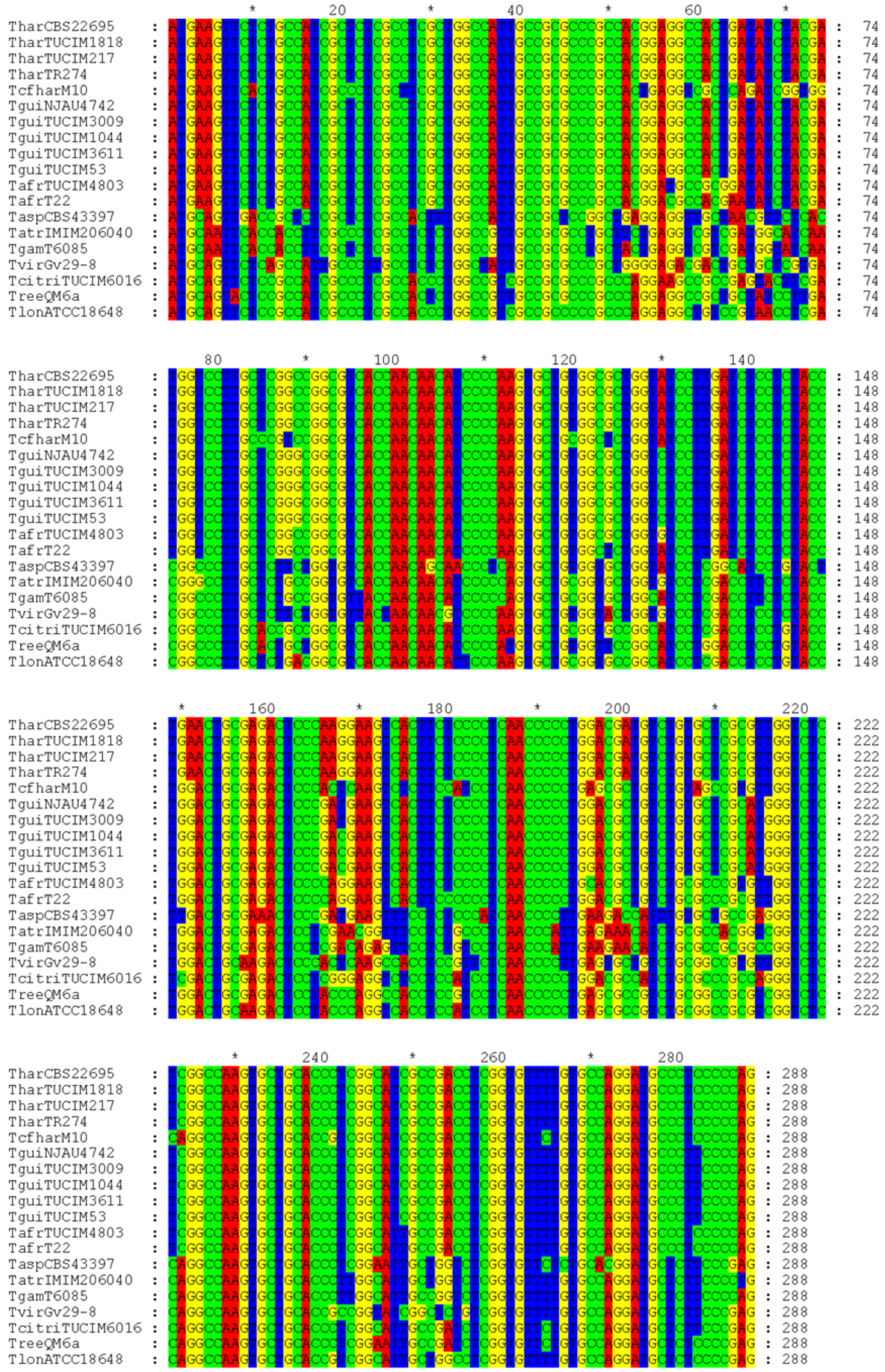
TharCBS22695
TharTUCIM1818
TharTUCIM217
TharTR274
TcfharM10
TguiNJAU4742
TguiTUCIM3009
TguiTUCIM1044
TguiTUCIM3611
TguiTUCIM53
TafrTUCIM4803
TafrT22
TaspCBS43397
TatrIMIM206040
TgamT6085
TvirGv29-8
TcitriTUCIM6016
TreeQM6a
TlonATCC18648
20
40
60
74
80
100
120
140
148
160
180
200
220
222
240
260
280
288

b

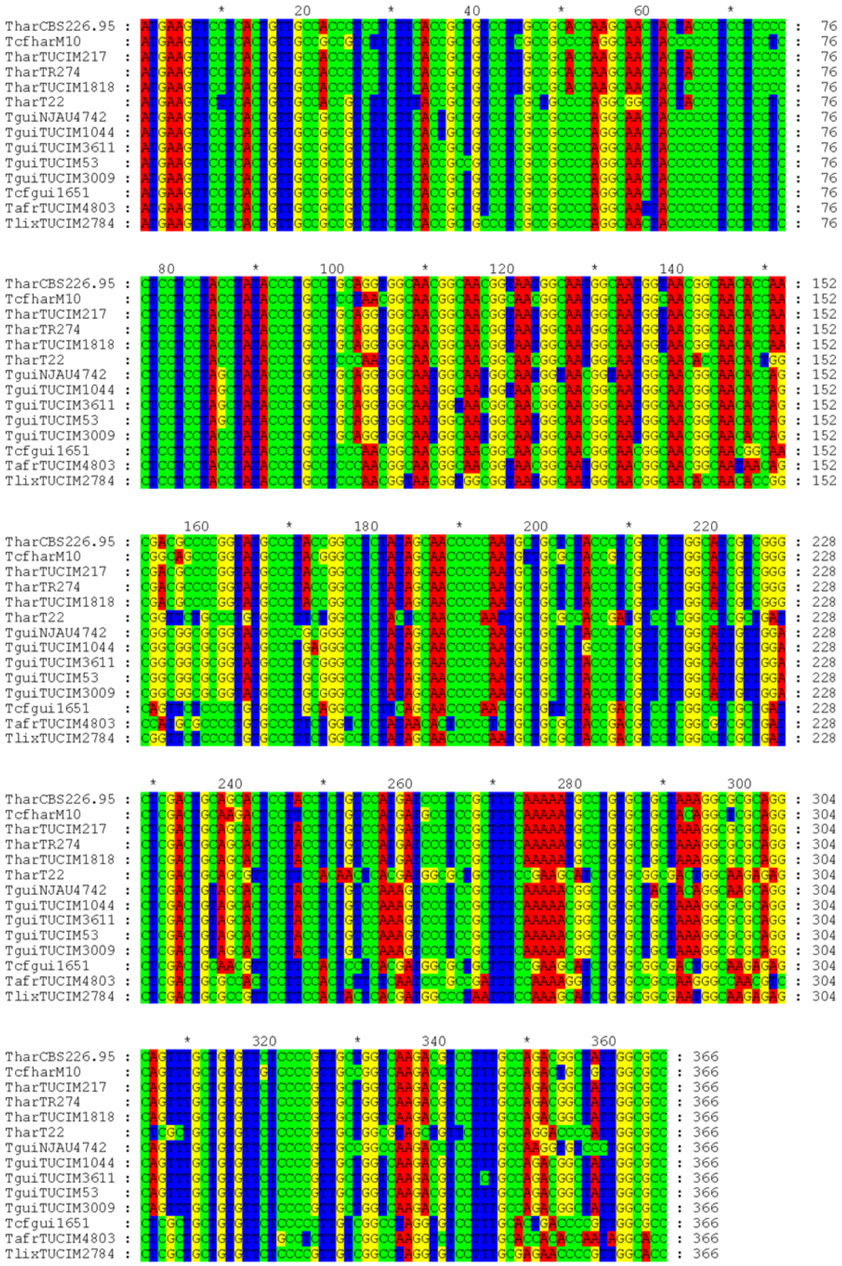

Fig. S9. Codon sequence alignment of *hfb4* (a) and *hfb10* (b) from *T. guizhouense*, *T. harzianum* and their closely-related species.

The alignment was performed by using Gendoc 1.5. Thar, *T. harzianum*; Tgui, *T. guizhouense*; Tcfhar, *T.* cf. *harzianum*; Tcfgui, *T.* cf. *guizhouense*; Tafr, *T. afroharzianum*; Tasp, *T. asperellum*; Tatr, *T. atroviride*; Tgam, *T. gamsii*; Tvir, *T. virens*; Tcitri, *T. citrinoviride*; Tlon, *T. longibrachiatum*; Tree, *T. reesei*. TUCIM, TU Collection of Industrial Microorganisms, Vienna, Austria.

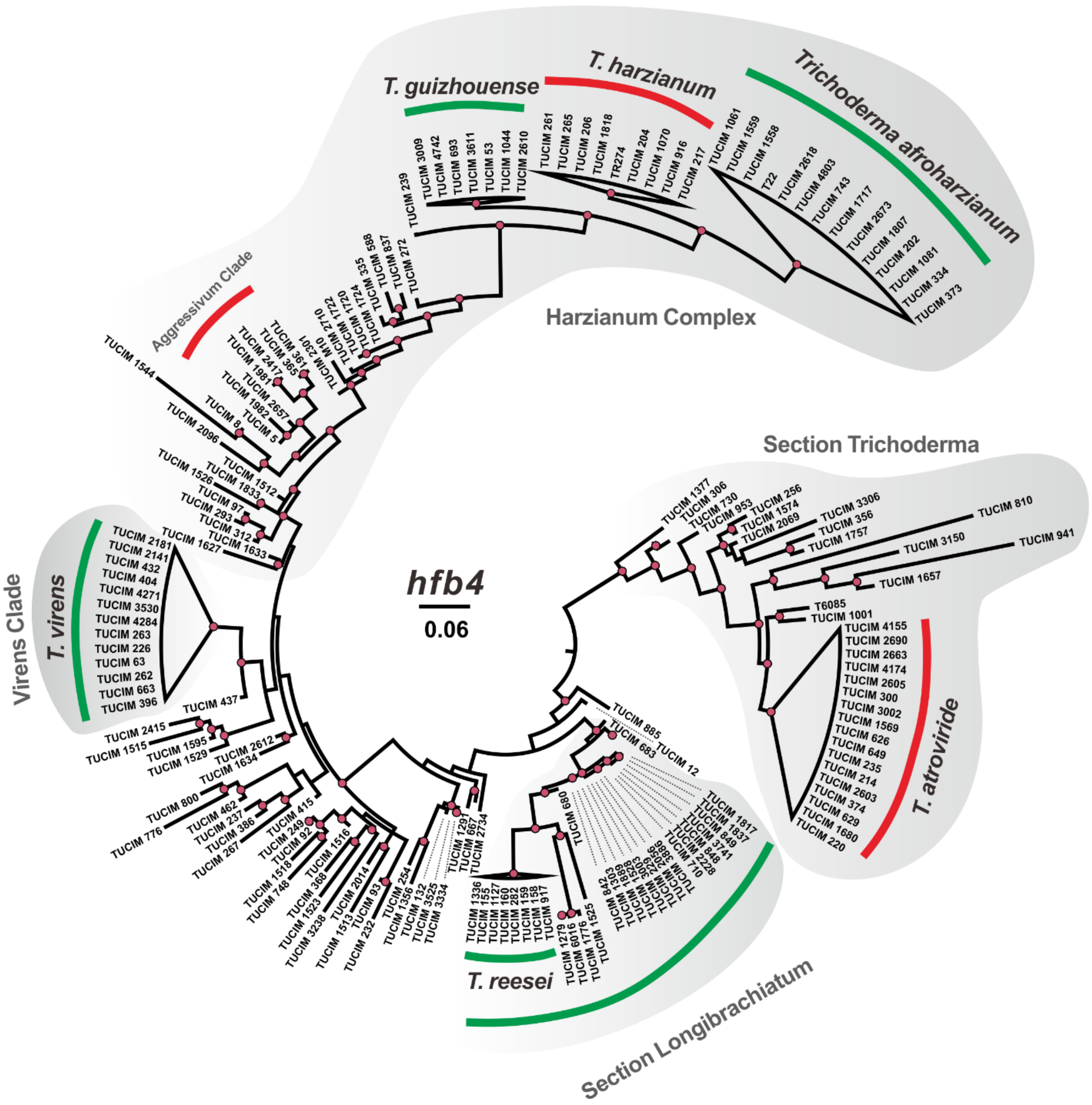

Fig. S10. Maximum likelihood phylogram of the *hfb4* gene from 170 strains belonging to the *Trichoderma* genus.

The phylogram was constructed by IQ-TREE 1.6.12 (Nguyen et al 2015). Red circles above nodes indicate IQTree ultrafast bootstrap support values >60. Lineages evolving under directional (positive) and stabilizing (purifying) selection pressure were respectively marked with red and green lines. The 170 *hfb4* sequences were obtained by sequencing the strains deposited in the TU Collection of Industrial Microorganisms (TUCIM) database with additional sequences retrieved from the genome-available strains, including *T. harzianum* TR274, *T. afroharzianum* T22, *T.* cf. *harzianum* M10 and *T. gamsii* T6085, in JGI database. Sequences deposited in the NCBI GenBank database accession numbers ranging from SAMN12793687 to SAMN12793837.

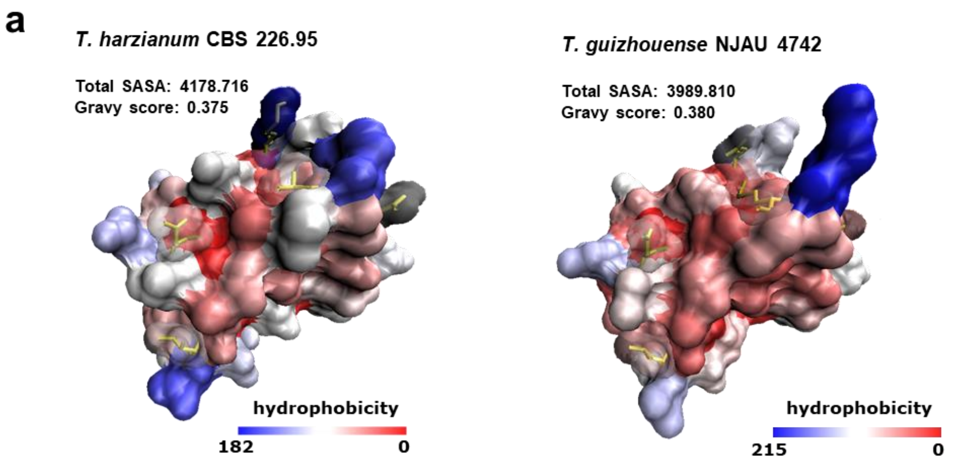

b

```
                                    *        20         *
TharCBS22695 : MKFSAIALFASLAIAAPATEATQEHQFDIYDGPCSA :  36
TguiNJAU4742 : MKFSAIALFASLAIAAPATEATQEHQFDIYDGPCSG :  36

                  40         *        60         *
TharCBS22695 : GVTNNIPKCCGAGILDLLYLNCETPKEVTSPLNPLD :  72
TguiNJAU4742 : GVTNNIPKCCGAGLLDLLYLDCETPDEVTSPLNPLD :  72

                      80         *        100
TharCBS22695 : DVCARVGLSAKCCTLGIADLGVLCQDALPQ : 102
TguiNJAU4742 : AVCARMGLSAKCCTLGIADLGVLCQDALPQ : 102
```

Fig. S11. Homology modelling (a) and protein sequence alignment (b) of HFB4 from *T. guizhouense* NJAU 4742 and *T. harzianum* CBS 226.95.

The homology models were generated by Modeller 9v15 (Webb and Sali 2016), without the signal peptide sequence, based on the structure of HFB2 (PDB ID: 2B97) from *T. reesei*. Residues A36G, I50L, N57D, K62D, D73A and V78M were different from *T. harzianum* CBS 226.95 and *T. guizhouense* NJAU 4742 (see the alignment in **b**). Five of these residues (I50L, N57D, K62D, D73A and V78M) were found to locate on the surface of the protein. Overall hydrophobicity was increased in $_{\mathrm{Tg}}$HFB4 relative to $_{\mathrm{Th}}$HFB4. Total solvent-accessible surface area (SASA) was calculated by using VMD with 1.4 Angstrom probe radius. Sequence based hydrophobicity was also calculated with Gravy calculator and showed the similar results, indicating that the hydrophobicity difference was most likely due to the mutation of residue 73 (D) in $_{\mathrm{Th}}$HFB4 where a charged residue changed to a hydrophobic residue (A) in $_{\mathrm{Tg}}$HFB4. TharCBS22695 and TguiNJAU4742 represent *T. harzianum* CBS 226.95 and *T. guizhouense* NJAU 4742, respectively, in **b**.

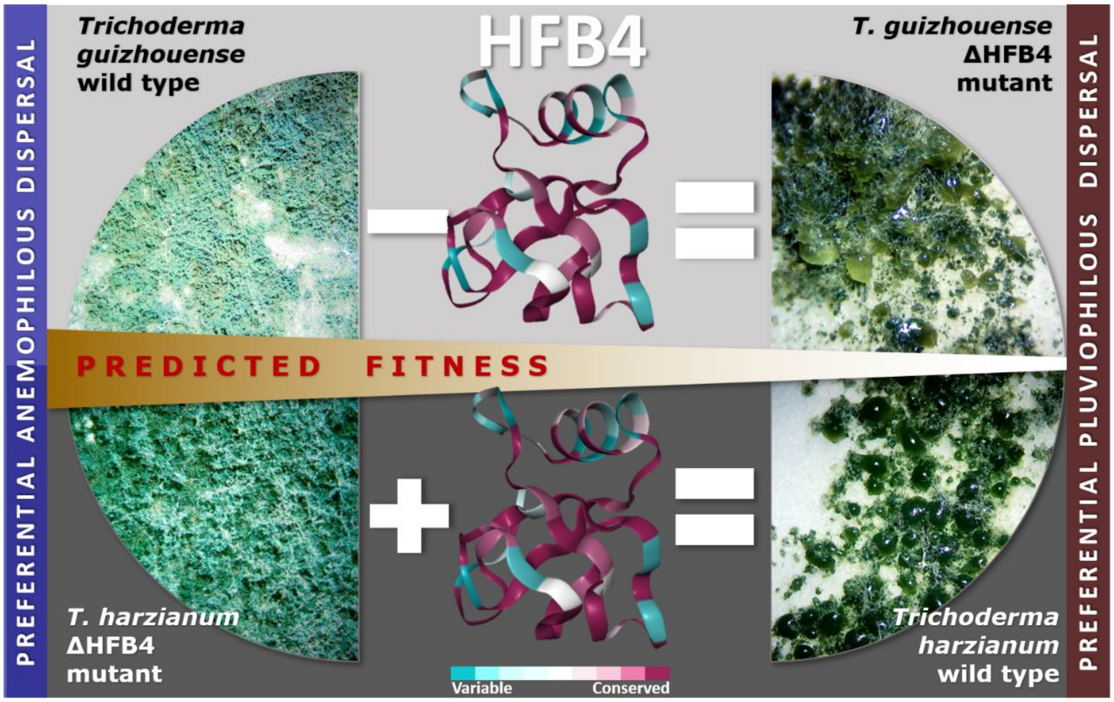

Fig. S12. Impact of the HFB4-encoding gene in *T. harzianum* and *T. guizhouense* on fitness-related parameters and putative preferential dispersal modes of these fungi.

The evolutionary conservation of amino acid sites on putative HFB4 models were estimated using the ConSurf tool (Ashkenazy et al. 2016) based on the model of HFB2 of T. reesei (PDB ID: 2B97) and the multiple sequence alignment (MSA) for the Harzianum Clade as provided in Supplementary Information S9a.

Dataset S1. (separate file). Raw data regarding fungal growth and reproduction potential.

The fungal growth of each strain grown on 95 carbon sources and on water in FF Biolog Microplates was shown by the values of O.D.750 nm at 8, 12, 18,24, 36, 48, 60, 72, 84, 96, 120, 144 and168 hours post inoculation, and the reproduction potential (including aerial hypha coverage and conidia abundance) was estimated by using the REproduction Potential Artificial INTellegence assay (REPAINT, see the main text for the description) on each carbon source at 72, 96, 120, 144 and168 hours post inoculation.

References

Ashkenazy H, Abadi S, Martz E, Chay O, Mayrose I, Pupko T et al (2016). ConSurf 2016: an improved methodology to estimate and visualize evolutionary conservation in macromolecules. Nucleic Acids Res **44:** W344-350.

Kubicek CP, Steindorff AS, Chenthamara K, Manganiello G, Henrissat B, Zhang J et al (2019). Evolution and comparative genomics of the most common *Trichoderma* species. BMC Genomics **20:** 485.

Nguyen LT, Schmidt HA, von Haeseler A, Minh BQ (2015). IQ-TREE: a fast and effective stochastic algorithm for estimating maximum-likelihood phylogenies. Mol Biol Evol **32:** 268-274.

Seiboth B, Karimi RA, Phatale PA, Linke R, Hartl L, Sauer DG et al (2012). The putative protein methyltransferase LAE1 controls cellulase gene expression in *Trichoderma reesei*. Mol Microbiol **84:** 1150-1164.

Uzbas F, Sezerman U, Hartl L, Kubicek CP, Seiboth B (2012). A homologous production system for *Trichoderma reesei* secreted proteins in a cellulase-free background. Appl Microbiol Biotechnol **93:** 1601-1608.

Webb B, Sali A (2016). Comparative protein structure modeling using MODELLER **54:** 5.6.1-5.6.37.
